# Pharmacological Inhibition of PPARy Boosts HIV Reactivation and Th17 Effector Functions, While Preventing Progeny Virion Release and de *novo* Infection

**DOI:** 10.20411/pai.v5i1.348

**Published:** 2020-09-30

**Authors:** Delphine Planas, Augustine Fert, Yuwei Zhang, Jean-Philippe Goulet, Jonathan Richard, Andrés Finzi, Maria Julia Ruiz, Laurence Raymond Marchand, Debashree Chatterjee, Huicheng Chen, Tomas Raul Wiche Salinas, Annie Gosselin, Eric A. Cohen, Jean-Pierre Routy, Nicolas Chomont, Petronela Ancuta

**Affiliations:** 1 Département de microbiologie, infectiologie et immunologie; Faculté de médecine; Université de Montréal; Montréal, Québec, Canada; 2 Centre de recherche du CHUM; Montréal, Québec, Canada; 3 Caprion; Montréal, Québec, Canada; 4 Institut de recherches cliniques de Montréal; Montréal, Québec, Canada; 5 Chronic Viral Illness Service; Division of Hematology; McGill University Health Centre-Glen site; Montreal, Québec, Canada

**Keywords:** HIV-1, ART, CD4+ T cells, Th17, PPARy, IL-21

## Abstract

The frequency and functions of Th17-polarized CCR6^+^RORyt^+^CD4^+^ T cells are rapidly compromised upon HIV infection and are not restored with long-term viral suppressive antiretroviral therapy (ART). In line with this, Th17 cells represent selective HIV-1 infection targets mainly at mucosal sites, with long-lived Th17 subsets carrying replication-competent HIV-DNA during ART. Therefore, novel Th17-specific therapeutic interventions are needed as a supplement of ART to reach the goal of HIV remission/cure. Th17 cells express high levels of *peroxisome proliferator-activated receptor gamma* (PPARy), which acts as a transcriptional repressor of the HIV provirus and the *rorc* gene, which encodes for the Th17-specific master regulator RORyt. Thus, we hypothesized that the pharmacological inhibition of PPARy will facilitate HIV reservoir reactivation while enhancing Th17 effector functions. Consistent with this prediction, the PPARy antagonist T0070907 significantly increased HIV transcription (cell-associated HIV-RNA) and RORyt-mediated Th17 effector functions (IL-17A). Unexpectedly, the PPARy antagonism limited HIV outgrowth from cells of ART-treated people living with HIV (PLWH), as well as HIV replication *in vitro*. Mechanistically, PPARy inhibition in CCR6^+^CD4^+^ T cells induced the upregulation of transcripts linked to Th17-polarisation (RORyt, STAT3, BCL6 IL-17A/F, IL-21) and HIV transcription (NCOA1-3, CDK9, HTATIP2). Interestingly, several transcripts involved in HIV-restriction were upregulated (Caveolin-1, TRIM22, TRIM5α, BST2, miR-29), whereas HIV permissiveness transcripts were downregulated (CCR5, furin), consistent with the decrease in HIV outgrowth/replication. Finally, PPARy inhibition increased intracellular HIV-p24 expression and prevented BST-2 downregulation on infected T cells, suggesting that progeny virion release is restricted by BST-2-dependent mechanisms. These results provide a strong rationale for considering PPARy antagonism as a novel strategy for HIV-reservoir purging and restoring Th17-mediated mucosal immunity in ART-treated PLWH.

## SIGNIFICANCE STATEMENT

The Th17-polarized CD4^+^ T cells are important players in mucosal immunity and their frequency/function are compromised during HIV infection despite viral-suppressive antiretroviral therapy (ART). Th17 cells are key HIV infection targets and contribute to viral reservoir persistence during ART. This raises the need for novel Th17-specific therapies. In this manuscript, we provide evidence that the pharmacological inhibition of PPARy, a documented repressor of the Th17 master regulator RORyt and HIV transcription, may represent a novel strategy toward Th17-mediated immunity restoration and HIV-reservoir purging in ART-treated PLWH.

## INTRODUCTION

Antiretroviral therapies (ART) efficiently control HIV-1 replication to undetectable plasma levels and have improved the life expectancy of people living with HIV (PLWH) [1-3]. However, ART does not cure HIV, with viral rebound occurring rapidly on treatment interruption [2, 4-6]. In addition, immunological dysregulations persist in ART-treated PLWH leading to an increased risk for non-AIDS co-morbidities such as cardiovascular disease [7] and neurocognitive impairment [8]. Therefore, additional therapeutic interventions to purge viral reservoirs and restore immuno-logical competence in ART-treated PLWH are needed [9].

In ART-treated PLWH, HIV reservoirs persist in a small fraction of long-lived memory CD4^+^ T-cells [3, 4, 10-12] and likely other cellular/anatomic reservoirs [13]. Studies by our group and others demonstrated that among CD4^+^ T cells, Th17-polarized cells are strategically located at portal sites of HIV/SIV entry and efficiently support integrative HIV infection [14-16]. Subsequently, Th17 cells are depleted from the gut-associated lymphoid tissues during HIV/SIV infection, and their frequency is not restored with ART [14, 15]. This leads to dramatic alterations in mucosal barrier integrity, increased microbial translocation from the gut, and systemic immune activation [14, 15], all leading to non-AIDS co-morbidities [7, 8]. Although the depletion of mucosal Th17 cells is well-documented during HIV/SIV infection, a fraction of Th17 cells is long lived and enriched in HIV-DNA in the blood and colon of ART-treated PLWH [14, 15]. The key role played by Th17 cells in mucosal homeostasis, their contribution to HIV persistence, as well as the deleterious consequence of their paucity in ART-treated PLWH, indicate that the design of novel Th17-specific therapeutic strategies is needed for HIV remission/cure [14, 15].

Th17 cells are distinguished from the other CD4^+^ T-cell subsets by a unique transcriptional signature that includes multiple HIV permissiveness factors (eg, CCR5, NF-κB, mTOR, NFATC2IP), the lack of anti-HIV defense mechanisms [14, 15], as well as the *peroxisome proliferator-activated receptor gamma* (PPARy) [17-20]. PPARy is an intrinsic negative regulator of NF-κB (21) and an inhibitor of HIV transcription [17, 22-24]. PPARy is a member of the PPAR subfamily of ligand-dependent non-steroid nuclear receptors; PPARy forms an obligatory heterodimer with *retinoic X receptor* (RXR) and binds onto PPAR responsive elements (PPREs) expressed on the promoters/regulatory regions of specific genes, thus functioning as a transcriptional repressor or activator [25, 26]. PPARy is expressed by multiple immune and non-immune cells and acts as a lipid sensor that controls the expression of numerous genes involved in lipid/glucose metabolism. Natural and synthetic PPARy agonists have been documented to regulate metabolic/inflammatory processes [26-29], in part via the mTOR activation pathway [30]. It is noteworthy that PPREs are present in the HIV long terminal repeat (LTR) region, indicating that PPARy participates directly in the negative regulation of HIV transcription [31]. Increasing evidence supports a role of PPARy in the regulation of adaptive immunity by acting on T-cell proliferation and differentiation [27, 29, 32-34]. Of particular importance, it was reported that PPARy inhibits Th17 effector functions by the transcriptional repression of RORyt [32, 34], the master regulator of Th17 differentiation [14, 15].

Clinical trials were previously performed using PPARy agonists/activators, for example, rosiglita-zone (RGZ) for treating the lypodystrophy caused by specific classes of antiretroviral drugs [35], as well as metabolic syndrome and inflammation in HIV-infected individuals [36-39]. However, to our knowledge, no clinical trials were performed using PPARy targeting drugs in the context of HIV cure/remission strategies. Although the PPARy activation blocks HIV replication in primary T cells [17], with PPARy agonists being expected to promote deep latency, studies in SIV-infected rhesus macaques demonstrated that hematopoietic alterations caused by Nef are dependent on the PPARy activation and are mimicked by the PPARy agonist RGZ [40]. Based on this evidence, Prost *et al.* proposed that PPARy inhibition may be more appropriate to counteract hematopoietic alterations caused by HIV/SIV infections [40] and emphasized the need for the development of clinically advanced PPARy antagonists [41]. Of particular importance, the pharmacological inhibition of PPARy may promote HIV reservoir reactivation, in a manner similar to that of currently tested latency reversing agents (LRA) [42, 43]. This scenario is supported by our previous studies demonstrating that RNA interference against PPARy results in increased viral replication on exposure to wild type and single round VSV-G/HIV [17].

In this study, we investigated the effect of PPARy pharmacological inhibition on HIV reservoir reactivation and immune function restoration in Th17 cells, a subset enriched in PPARy mRNA and protein [17, 18]. Our results demonstrate that the PPARy antagonism increased both HIV transcription and RORyt-mediated Th17 effector functions, such as IL-17A and IL-21, in CD4^+^ T cells from ART-treated PLWH. Of note, IL-21 is a signature-cytokine for follicular helper T-cells (Tfh) [33] that is also key for Th17 survival [14] and has demonstrated antiviral activity *in vitro* [44] and in non-human primate models [45, 46]. Unexpectedly, the PPARy antagonism limited viral outgrowth in CD4^+^ T cells of ART-treated PLWH *ex vivo*, as well as on HIV infection *in vitro*. The unique combination of these immunological and virological features provides a strong rationale for considering the pharmacological inhibition of PPARy for HIV cure/remission strategies.

## MATERIALS AND METHODS

### Study participants

PLWH receiving viral-suppressive ART (Table 1) and HIV-individuals (n=15 males; n=2 females) were recruited at the Montreal Chest Institute, McGill University Health Centre and Centre Hospitalier de l'Université de Montréal (CHUM) in Montreal, Quebec, Canada. Large quantities of PBMCs (10^9^–10^10^ cells) were collected by leukapheresis, as previously described [19, 20].

**Table 1.** Clinical parameters of ART-treated PLWH study participants.

Patient ID	Sex	CD4 count#	CD8 count#	Plasma viral load&	Time since infection*	ART	Time on ART*
**ART #1**	M	398	775	<40	154	Complera	26
**ART #2**	M	841	1,322	<40	150	Sustiva/Truvada	138
**ART #3**	M	796	399	<40	8	Stribild	6
**ART #4**	M	581	1,060	<40	96	Sustiva/Truvada	5
**ART #5**	M	391	620	50	165	Kivexa/Delavirdine	54
**ART #6**	M	318	431	<40	149	Kivexa/Delavirdine	44
**ART #7**	M	514	568	<40	16	Tivicay/Truvada	6
**ART #8**	M	775	1,000	<40	74	Complera	19
**ART #9**	M	459	545	<40	189	Truvada/Raltegravir	>12
**ART #10**	F	616	330	<40	186	Viracept/Truvada	34
**ART #11**	M	542	803	<40	13	Stribild	12
**ART #12**	M	458	899	<40	201	Truvada/Viramune	200
**ART #13**	M	908	854	<40	89	Stribild	72
**ART #14**	F	833	445	<40	213	Viracept/Truvada	60
**ART #15**	M	546	1,116	<40	408	Atripla	372

# *cells/µl*;

& HIV-RNA copies per ml plasma;

* months; ART, antiretroviral therapy; M, male; F, female

### Ethics statement

This study, using PBMCs from HIV-uninfected and HIV-infected study participants was conducted in compliance with the principles included in the Declaration of Helsinki. This study received approval from the Institutional Review Board (IRB) of the McGill University Health Centre and the IRB of the CHUM-Research Centre, Montreal, Quebec, Canada. All participants signed a written informed consent and agreed with the publication of the results generated using their biological samples.

### Drugs

The following drugs were used: T0070907 (T007; 2-Chloro-5-nitro-*N*-4-pyridinylbenzamide; Tocris, Cayman Chemical, Michigan, USA); rosiglitazone (RGZ; Cayman Chemical, Michigan, USA); Saquinavir, and Raltegravir (NIH AIDS Reagent Program, Maryland, USA).

### Flow cytometry analysis

The fluorochrome-conjugated antibodies used for polychromatic flow cytometry are listed in Supplemental Table 3. A viability dye (Molecular Probes^®^ LIVE/DEAD^®^ Fixable Dead Cell Stain Kits, Invitrogen) was used to exclude dead cells. Intracellular staining was performed using Fixation/Permeabilization Solution Kit (BD). Cells were analyzed using an LSRII cytometer, Diva version 6 (BD Biosciences, San Jose, CA), and FlowJo version 10.0.6 (Tree Star, Inc). Flow cytometry gates were defined using the fluorescence minus one (FMO) strategy [19, 20].

### Cell sorting

Total and memory CD4^+^ T cells were enriched from PBMCs by negative selection using magnetic beads (magnetic-activated cell sorting [MACS], Miltenyi), with a purity of >95%, as previously described [19, 20]. Highly pure CCR6^+^/CCR6^-^ T cells were sorted by FACS using antibodies listed in Supplemental Table 3, as previously reported by our group [19, 20].

### Viral outgrow assay

A viral outgrowth assay (VOA) was performed using a protocol previously established by our group [19, 20]. Briefly, total memory CD4^+^ T cells isolated by MACS from PBMCs of PLWH receiving viral-suppressive ART (PLWH+ART) were cultured (RPMI1640, 10% FBS, 1% antibiotics) at 1x10^6^ cells/mL/well in 48-well plates in the presence of immobilized CD3 and soluble CD28 antibodies (1 µg/mL) for up to 12 days. At day 3, cells were washed, split into 2 new wells, and cultured with IL-2 (5 ng/mL). At days 6 and 9, cells from each well were split into 2 new wells, and media was refreshed. Supernatants were collected at days 3, 6, 9, and 12 for HIV-p24 and cytokine quantification by ELISA. At day 12, cells were stimulated with PMA (50 ng/mL) and Ionomycin (1ug/mL) in the presence of Brefeldin A (5 ug/mL) for 5 hours and used for the intra-cellular detection of HIV-p24, IL-17A, and IFN-y by flow cytometry after staining with specific antibodies (Supplemental Table 3).

### Quantification of cell-associated HIV-RNA and HIV-DNA

Cell-associated (CA) RNA and DNA was dually extracted from cell pellets (polled 5-6 replicates of 1x10^6^ cells/experimental condition) using the AllPrep DNA/RNA Mini Kit (Qiagen), according to the manufacturer's instructions. The quality (260 nm/280 nm ratio) and quantity of RNA/DNA collected were evaluated by Nanodrop.

CA LTR-Gag HIV-RNA (CA HIV-RNA) levels were quantified by 1-step real-time RT-PCR using specific external/internal primers and taqman probes (Supplemental Table 4a) and classical RTPCR/PCR amplification conditions. The amplified products from the first PCR (ProFlex PCR System 9700; Applied Biosystems) were diluted 10 x in molecular grade water and used as templates in second nested real-time PCR amplifications (RotorGene instrument, Qiagen). For the CA LTR-Gag HIV-RNA (unspliced), standards were generated using plasmid-based transcription *in vitro* (MEGAscript™ T7 Transcription Kit, ThermoFisher).

To normalize HIV-RNA to HIV-DNA on matched samples, levels of CA Gag HIV-DNA were quantified by ultrasensitive nested real-time PCR using the same primers and Taqman probe used for the CA HIV-RNA quantification (Table 4a). To normalize the HIV-DNA levels per number of cells, the CD3 gene was concomitantly amplified using specific external/internal primers and Taqman probes (Supplemental Table 4b), as previously described [19, 20]. ACH2 cells carrying 1 copy of integrated HIV-DNA per cell (The National Institutes of Health AIDS Reagent Program) were used for the standard curve.

### Quantification of cell-free HIV RNA

The quantification of cell-free HIV-RNA was performed as previously reported [47]. To enrich in HIV virions, 5 mL aliquots of cell culture supernatants were centrifuged at 25,000*g* for 90 minutes. Pelleted virions (in 140 µL supernatant) were used for total RNA isolation using the QIAamp Viral RNA Mini Kit (Qiagen; final elution in 60 µL). The extracted RNA was first subjected to DNase (Invitrogen) treatment. HIV-RNA quantification was performed as described above. HIV-RNA quantification was performed in triplicates (using 17 µL eluted total RNA/test), as described above. Results are expressed as the number of HIV-RNA copies per reaction (equivalent of 5 mL cell culture supernatant per test). Standards were generated using RNA extracted from ACH2-culture supernatant. All measures were performed in triplicate.

### HIV infection *in vitro*

T cells were activated with CD3/CD28 antibodies (1 µ g/mL), exposed to the replication-competent transmitted/founder (T/F) strain HIV _THRO_ (NIH AIDS Reagent Program) [48], and viral replication monitored by ELISA, as previously described [19, 20]. Infected cells were cultured with IL-2 (5 ng/mL), in the presence or absence of T0070907 (1 µM, 5 µM, 10 µM) or RGZ (50 µM). In parallel, experiments were performed with single round VSV-G-pseudotyped HIV (VSV-G/HIV; NL4.3 backbone, *env*-, *gfp* in place of *nef*) [49]. The viral stocks were produced by transfection of 293T cells, as previously described [17, 18].

### HIV integration

Integrated HIV-DNA was quantified by ultrasensitive nested real-time PCR in cell lysates (10^5^ cells/test in triplicate; detection limit: 3 HIV-DNA copies/test), with normalization relative to CD3 copy numbers (2 CD3 copies per single cell), as previously described [12, 19, 20], using specific primers and FRET probes (Supplemental Tables c-d).

### Real-time RT-PCR for quantification of cellular transcripts

Total RNA was isolated using the RNeasy Kit (Qiagen) and quantified using the Pearl nanophotometer (Implen). One step SYBR Green real-time RT-PCR (Qiagen) was carried out in a Light-Cycler 480 II (Roche) according to the manufacturer's recommendations, as we previously reported [17, 18]. QuantiTect Primer Assays were purchased from Qiagen. The expression of each gene was normalized relative to 28S rRNA levels. Amplifications were performed in triplicate on 70 ng RNA/test for target genes and 2 ng RNA/test for 28S rRNA.

### Genome-wide RNA-sequencing and analysis

Genome-wide transcriptional profiling was performed on total RNA by Genome Québec (Montreal, Québec, Canada) using the Illumina RNA-Sequencing model HiSeq 4000 PE100. Briefly, the paired-end sequencing reads were aligned to coding and non-coding transcripts from Homo Sapiens database GRCh 37 version75 and quantified using the kallisto software version 0.44.0 [50]. The entire RNA-Sequencing data set and the technical information requested by Minimum Information About a Microarray Experiment (MIAME) are available at the Gene Expression Omnibus database under accession **GSE128121**. One-way ANOVA analysis identified differentially expressed genes based on *P* values (*P*<0.05) or adjusted *P* values (adj. *P*<0.05) and/or fold-change (FC, cutoff 1.3). Statistical analyses were performed using R version 3.5.1. Differential expression analysis was performed using the limma Bioconductor package [51] (version 3.38.3) on the log_2_-counts per million (logCPM) transformed transcript-level data. Gene set enrichment analysis was performed using the GSVA method [52] (package version 1.30.0) on the logCPM data using a Gaussian cumulative distribution function.

### Statistics

All statistical analyses were performed using the Prism 8 (GraphPad software). Specifications on the statistical test used are included on the graphs and Figure legends. *P* values are indicated on the graphs with statistical significance as follows: *P < 0.05; **P < 0.01; ***P < 0.001; ****P < 0.0001

## RESULTS

### PPARy inhibition increases IL-17A and HIV transcription but reduces viral production and release in CD4^+^ T cells of ART-treated PLWH.

**Figure 1.**
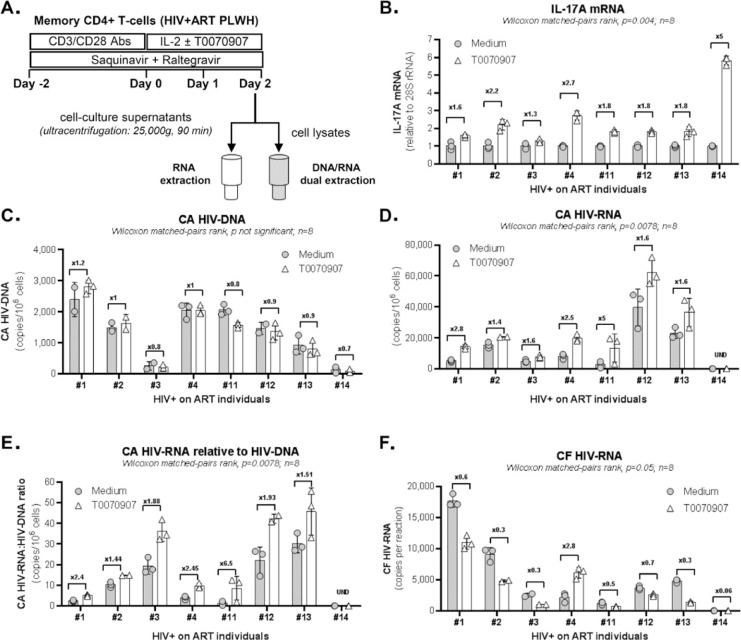
**The** PPARy **antagonist T0070907 increases HIV and IL-17A transcription but inhibits viral release from memory CD4^+^ T cells of ART-treated PLWH. (A)** Shown is the experimental flow chart. Briefly, memory CD4^+^ T cells of ART-treated PLWH (Table 1, n=8) were activated by CD3/CD28 for 2 days in the presence of ARVs (Saquinavir 5μM; Raltegravir 200nM) to limit cell-to-cell virion spreading, washed and further cultured with ARVs in the presence or the absence of T0070907 (10μM) for other 48 hours. DMSO (1 μL/mL; identified as Medium) was used as a control. Total RNA and DNA levels were dually extracted from cell pellets and total RNA was extracted from cell culture supernatants. **(B)** IL-17A mRNA was quantified by real-time RT-PCR and normalized to 28S rRNA levels. **(C)** Cell-associated (CA) HIV-DNA (Gag primers) were quantified by nested real-time PCR and normalized per 10^6^ cells (2 copies CD3-DNA per cell). **(D-E)** CA HIV-RNA (unspliced, Gag primers) levels were quantified by nested real-time RT-PCR and normalized per 10^6^ cells **(D)** and HIV-DNA/10^6^ cells (**E**) using results from panel **C**. (**F)** Cell-free (CF) HIV-RNA (Gag primers) copies were quantified by nested real-time RT-PCR in RNA extracted from cell culture supernatants. Each symbol represents 1 experimental replicate (mean±SD). The Wilcoxon matched-pairs signed rank test *P*-values and the fold change (FC) ratios between medium and T0070907 are indicated on the graphs.

**Supplementary Figure 1.**
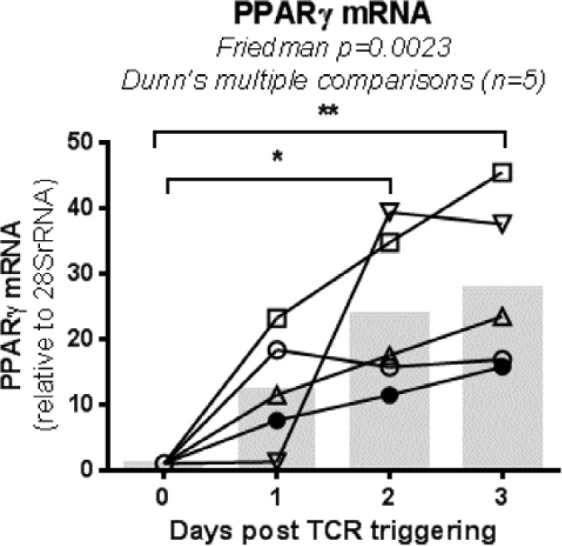
PPARy mRNA express is introduced by TCR triggering. RNA was extracted from memory CD4+ T cells from HIV-uninfected individuals (n=5), either freshly-isolated (Day 0) or stimulated CD3/CD28 for 1, 2, or 3 days. PPARy mRNA levels were quantified by RT-PCR (triplicates, 60 ng RNA/test). Each symbol represents results generated with cells from a different individual. Grey bars indicate median PPARy mRNA levels for n=5. Friedman test *P*-values and Dunn's multiple comparisons are indicated on the graph.

**Supplementary Figure 2.**
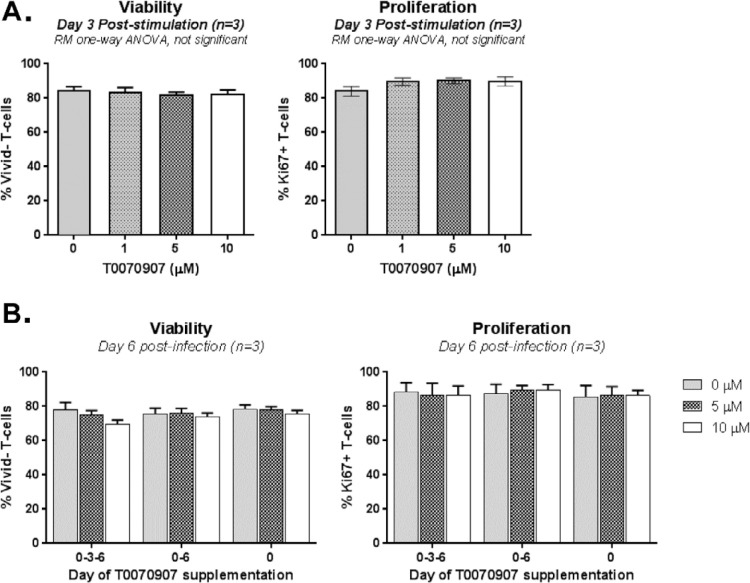
**Effects of T0070907 of CD4+ T-cell viability and proliferation.** (A) Memory CD4^+^ T cells isolated from HIV-uninfected individuals were 11 stimulated by CD3/CD28 and cultured in the presence/absence of different doses of 12 T0070907 (1, 5, and 10μM) for 3 days, with T0070907 being administered only once. 13 Cells were stained with a viability dye (Vivid), and then intranuclear staining was 14 performed with Ki67 antibodies; Ki67, a marker for cell cycle progression, was used as a 15 surrogate marker of cell proliferation. Shown is cell viability (Vivid-cells, left panel) 16 and proliferation (Ki67+ cells, right panel) measured by flow cytometry with cells from 17 n=3 individuals. (B) Memory CD4+ T cells isolated from HIV-uninfected individuals 18 were stimulated by CD3/CD28, infected with HIVTHRO (as described in Figure 3 legend), 19 and cultured in the presence or the absence of T0070907 (5 and 10 μM). To determine 20 the effect of single versus multiple T0070907 doses on cell viability/proliferation, 21 T0070907 was administered either once at day 0 post-infection (0), twice at days 0 and 6 post-infection (0-6), or every 3 days post-infection (0-3-6). Shown are mean±SD of values obtained with cells from n=3 individuals.

We hypothesized that PPARy pharmacological inhibition promotes both HIV reservoir reactivation and immune function restoration in Th17 cells. To test this hypothesis, we characterized the effects of the well-characterized PPARy antagonist T0070907 [53] in memory CD4^+^ T cells from ART-treated PLWH (Table 1, n=8) (Figure 1A). Cells were stimulated with CD3/CD28 antibodies for 2 days to induce HIV optimal outgrowth [47] and PPARy expression (Supplemental Figure 1) [17]; cells were further cultured in the presence/absence of T0070907 for 2 additional days. To study the post-integration steps of viral replication (ie, transcription, virion production and release) while preventing novel infection *in vitro*, experiments were performed in the presence of the antiretroviral drugs (ARV) Saquinavir and Raltegravir (Figure 1A). Preliminary experiments allowed the identification of an optimal T0070907 concentration (ie, 10µM) that upregulates IL-17A production without affecting cell viability/proliferation (Supplemental Figure 2A). As expected, exposure to T0070907 resulted in a significant increase of IL-17A mRNA levels (Figure 1B). Upon this short-term stimulation/culture *in vitro*, CA HIV-DNA levels remained similar in T cells cultured with or without T0070907 (Figure 1C), consistent with the well-established stability of HIV-DNA reservoirs [4, 10]. Nevertheless, exposure to T0070907 significantly increased absolute CA HIV-RNA levels, as well as CA HIV-RNA:HIV-DNA ratios (Figure 1D-E), indicating that the drug boosted the TCR-mediated HIV transcription. Unexpectedly, cell-free HIV-RNA levels were significantly reduced by T0070907 in 7 of 8 donors (Figure 1F), indicative of a post-transcriptional block in virion production/release. Thus, the PPARy antagonism overcomes the PPARy-mediated repression of RORyt and HIV transcription, but also modulates expression of other factors acting at the post transcriptional level, thus resulting in decreased *de novo* production and release of viral particles.

**Figure 2.**
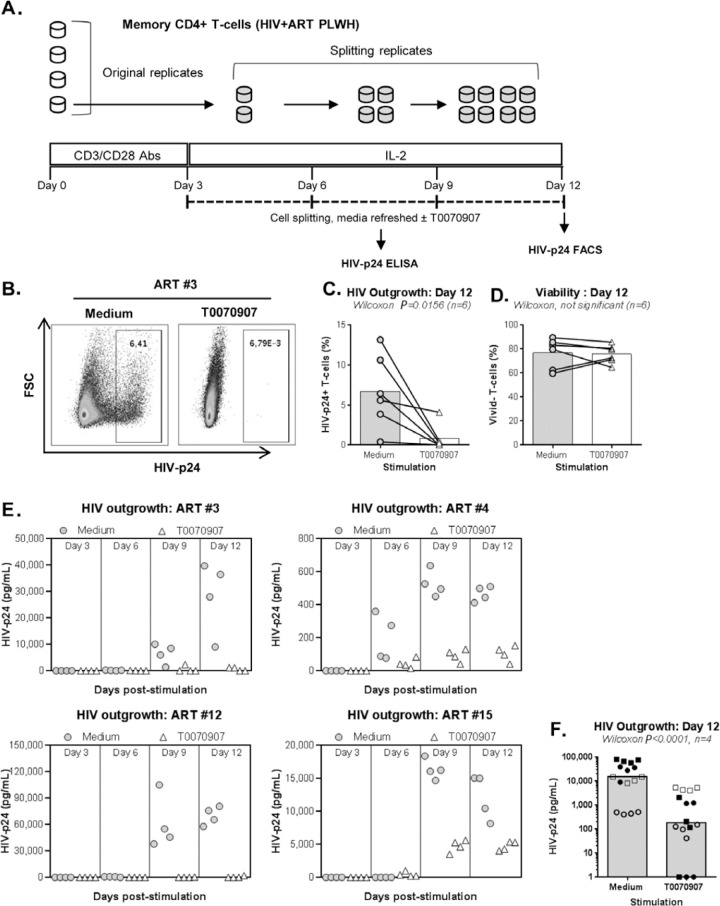
**T0070907 inhibits HIV outgrowth in memory CD4^+^ T cells of ART-treated PLWH. (A)** Shown is the experimental flow chart for the viral outgrowth assay (VOA) performed with memory CD4^+^ T cells of ART-treated PLWH. Briefly, cells cultured in 48-well plates (10^6^ cells/well) were activated with CD3/CD28 antibodies for 3 days, washed and cultured in the presence or the absence of T0070907 (10μM) up to 12 days. Cells were split into 2 new wells, supernatants collected and media refreshed every 3 days. At day 12, cells were stained with a viability dye and then intracellularly with HIV-p24 antibodies. **(B-D)** In a first set of experiments, the VOA was performed with one original replicate (10^6^ cells/well) at day 0 that generated 8 splitting replicates at day 12. Shown is **(B)** the intracellular HIV-p24 expression in cells pooled from the 8 splitting replicates at day 12 from one representative donor (ART #3), as well as statistical analysis of **(C)** intracellular HIV-p24 staining and **(D)** cell viability in n=6 ART-treated PLWH (Table 1; ART #3, #4, #5, #10, #12, and #15). **(E-F)** In another set of experiments, the VOA was performed in 4 original replicates of 10^6^ cells/well cultured at day 0 that each generated 8 splitting replicates at day 12. Shown are HIV-p24 levels in cell culture supernatant quantified in cell culture supernatant collected from the splitting replicates of each original replicate at days 3 (1 well), 6 (2 wells), 9 (4 wells), and 12 (8 wells) for each donor individually **(E)** and statistical analysis on n=4 ART-treated PLWH at day 12 **(F)** (Table 1; ART #3, #4, #12, and #15). Each symbol represents the median HIV-p24 value of 8 splitting replicate wells resulting from 1 original replicate (4 original replicates/donor), with grey circles for Medium and open triangles for T0070907 **(E)** and different symbols for each donor **(F)**. The Wilcoxon matched-pairs signed rank test *P*-values and the fold change (FC) ratios between medium and T0070907 are indicated on the graphs.

### PPARy antagonism inhibits HIV outgrowth from CD4+ T cells of ART-treated PLWH

Productive HIV replication is regulated at multiple post-transcriptional steps [1]. To further document the effect of PPARy antagonism on *de novo* HIV production, a VOA that monitors viral reservoir reactivation and cell-to-cell propagation in culture [19, 20] was performed (Figure 2A). To optimally detect replication-competent HIV, memory CD4^+^ T cells were isolated from PLWH receiving ART for >2 years (#5, #10, #12, and #15) and receiving ART <2 years (#3 and #4) (Table 1). In a first set of experiments, HIV outgrowth was measured by intracellular HIV-p24 staining at day 12 post-stimulation in cells from 8 splitting replicates merged together (generated from 1 original replicate). Results in Figure 2B-C demonstrate that the HIV outgrowth induced by CD3/CD28 triggering was significantly reduced in the presence of T0070907, with no significant impact on cell viability (Figure 2D). By merging the cells from the 8 identical replicates, it was possible to stimulate the cells with PMA/Ionomycin and monitor the expression of HIV-p24 in cells production IL-17A and/or IFN-y. Consistent with the well-documented Th17 cell permissiveness to HIV [14, 15], when the VOA was performed in the absence of T0070907, the highest frequency of infected cells was detected in Th17 (IL-17A^+^IFN-y^-^) and Th1Th17 (IL-17A^+^IFN-y^+^) cells; T0070907 reduced the frequency of HIV-p24^+^ but not IL-17A^+^ cells (data not shown). These results indicate the ability of T0070907 to limit HIV replication in Th17 cells without altering their effector functions.

Considering the stochastic distribution of HIV reservoirs, the VOA was performed again with cells from n=4 ART-treated PLWH (Table 1; ART #3, #4, #12, and #15), but this time using 4 original replicates of 10^6^ cells/well (Figure 2E-F) instead of 1 (Figure 2B-D). The HIV-p24 ELISA quantification was performed in cell culture supernatants collected at days 3, 6, 9, and 12 post-stimulation from all splitting replicates. Results in Figure 2E-F confirmed the capacity of T0070907 to inhibit HIV outgrowth.

**Supplementary Figure 3.**
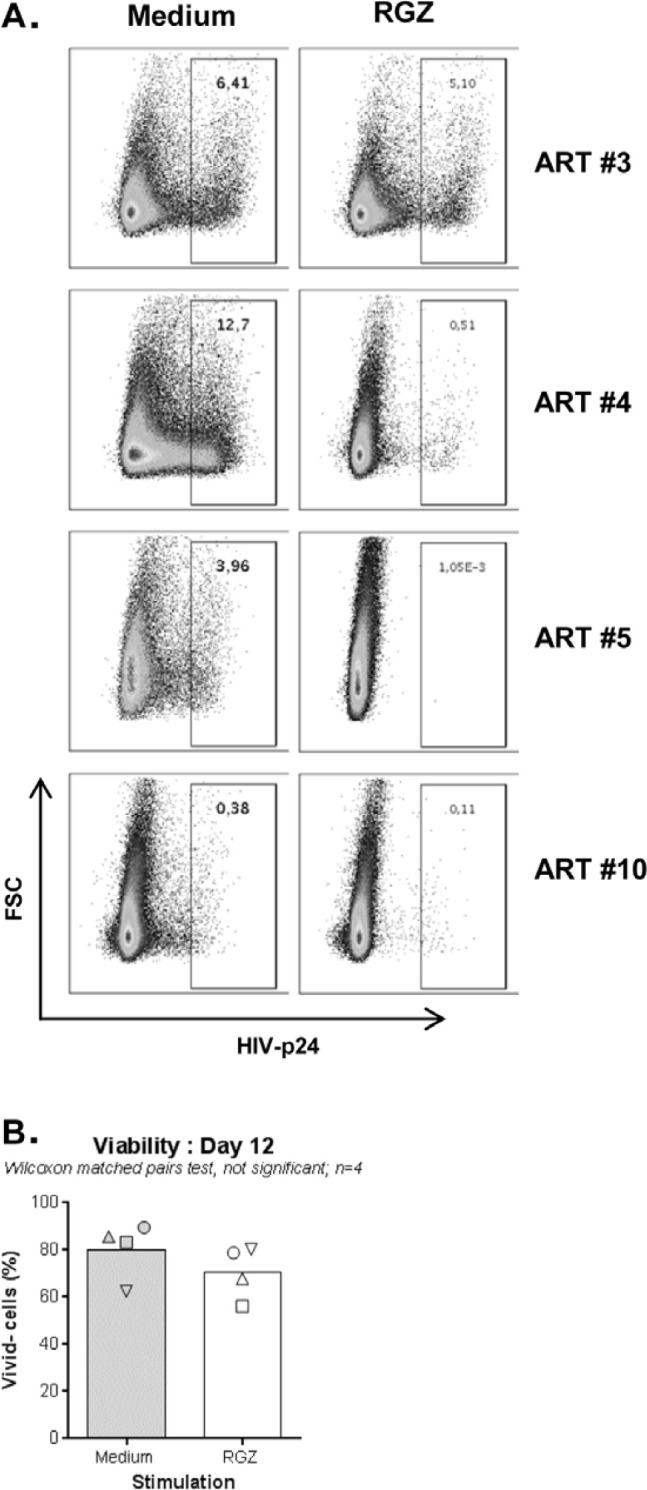
**RGZ limits HIV outgrowth in memory CD4+ T cells of ART-treated PLWH.** A VOA was performed with memory CD4+ T cells of ART treated PLWH (Table 1; ART #3, 4, 5, and 10) in the presence/absence of rosiglitazone (RGZ; 50 µM). At day 12 post-culture, cells were stained as in Figure 2B. Shown is the effect of RGZ on the intracellular expression of HIV-p24 in 4 different ART-treated PLWH (A), as well as the effect of RGZ exposure on cell viability (B). Wilcoxon matched-pairs signed rank test P-values are indicated on the graph in B.

Given the documented ability of RGZ in inhibiting HIV replication [17, 54] by repressing HIV transcription [31], we used RGZ as a control in this VOA. As expected, RGZ (50 µM, optimal dose previously identified [17]) inhibited viral outgrowth in cells of ART-treated PLWH (Table 1; ART #3, #4, #5, and #10) (Supplemental Figure 3A), with no significant effects on cell viability (Supplemental Figure 3B).

**Figure 3.**
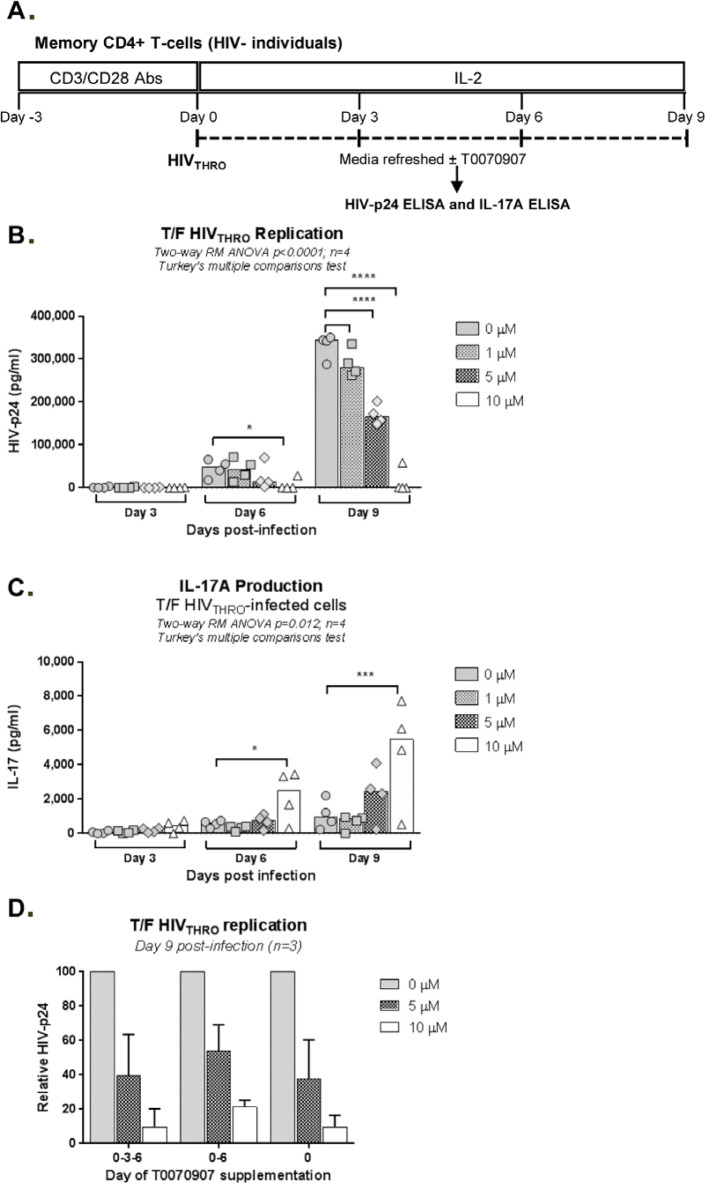
**T0070907 boosts IL-17A production and limits T/F HIV_THRO_ replication *in vitro* in a dose-dependent manner. (A)** Shown is the experimental flow chart. Briefly, memory CD4^+^ T cells isolated from HIV-uninfected individuals were stimulated by CD3/CD28 for 3 days. **(B-C)** Cells were exposed to T/F HIV_THRO_ strain (25 ng/10^6^ cells) and cultured in the presence of IL-2 (5 ng/ml) and in the presence/absence of T0070907 (1, 5, and 10µM) for up to 9 days, with media, IL-2 and/or T0070907 being refreshed every 3 days. Shown are HIV-p24 levels **(B)** and IL-17A **(C)** quantified by ELISA in cell culture supernatants at days 3, 6 and 9 post-infection (n=4). Each symbol represents 1 different donor, and bars represent median values. Two-way RM ANOVA *P*-values and Turkey's multiple comparisons are indicated on the graphs. **(D)** To determine the effect of single versus multiple T0070907 doses on HIV replication, in another set of experiments, infected cells were cultured in the presence of IL-2 and in the presence/absence of T0070907 (5 and 10µM), with T0070907 being administered either once at day 0 post-infection (0), twice at days 0 and 6 post-infection (0-6), or every 3 days post-infection (0-3-6). Shown are relative HIV-p24 levels quantified by ELISA in cell culture supernatants collected at day 9 post-infection (n=3).

Thus, the PPARy antagonism inhibits viral outgrowth by acting on viral replication steps downstream of transcription, steps that are important for *de novo* viral particle production and/or propagation and spread.

### PPARy inhibition reduces HIV replication *in vitro*

Considering the unexpected antiviral features of T0070907, we further investigated its ability to modulate HIV replication *in vitro*. For this, we used the transmitted/founder (T/F) strain THRO, documented to exhibit high virulence [55], using the experimental design depicted in Figure 3A. TCR-activated memory CD4^+^ T cells were infected with HIV_THRO_ and treated with T0070907 (1, 5, 10µM) for up to 9 days, with T0070907 being refreshed in the media every 3 days. Results indicate a dose-dependent effect of T0070907, with a significant increase in IL-17A production and a decrease in HIV replication observed at 10µM (Figure 3B-C), with no effects on cell viability and proliferation (Supplemental Figure 2A). In parallel, similar experiments were performed with T0070907 being added every 3 days versus once (day 0 post infection) or twice (day 0 and 6 post-infection). Results in Figure 3D clearly demonstrate that the antiviral effect of T0070907 is achieved with a single dose of T0070907 added immediately on infection. No effects on cell viability and proliferation were observed (Supplemental Figure 2B). This is indicative that PPARy inhibition during the early steps of infection allows a robust control of HIV spread in culture.

**Supplementary Figure 4.**
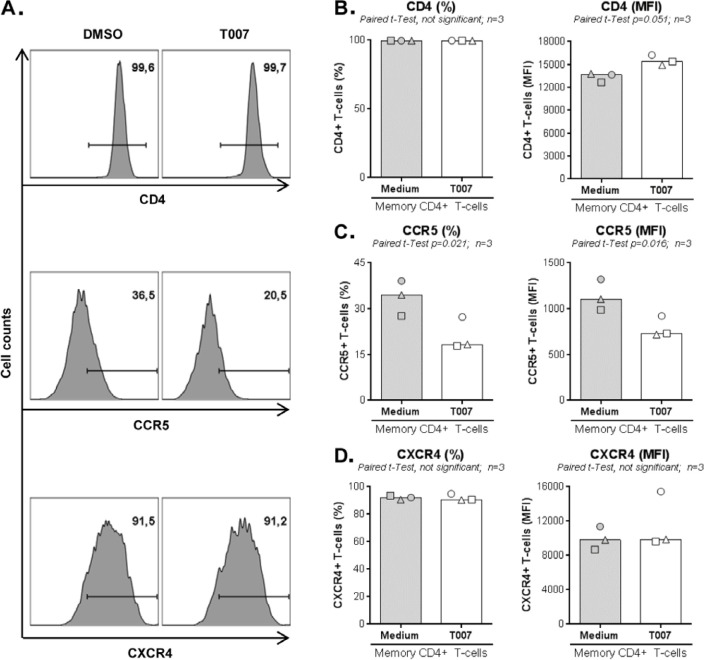
**T0070907 decreases CCR5 expression on memory CD4+ T cells.** Memory CD4+ T cells from HIV-uninfected individuals were stimulated by CD3/CD28 for 3 days and then cultured in the presence/absence of T0070907 (10 µM) for 2 additional days. The expression of HIV-1 receptor CD4 and co-receptors CCR5 and CXCR4 was measured by flow cytometry. Shown are histograms from 1 representative donor (A), and the statistical analyses of CD4 (B), CCR5 (C), and CXCR4 (D) expression (% and MFI) in 3 different donors. Each symbol represents 1 different donor and bars represent median values. Paired t-test values are indicated on the graphs.

To get insights into the mechanisms of T0070907 action, we investigated its effect on the expression of the HIV receptor CD4 and co-receptors CCR5/CXCR4. Although T0070907 did not change CD4 and CXCR4 expression, a significant decrease in CCR5 expression was observed (Supplemental Figure 4A-D). Thus, in addition to reducing viral production/release (Figure 1F), T0070907 also limits *de novo* infection in part by limiting CCR5-mediated HIV entry.

**Figure 4.**
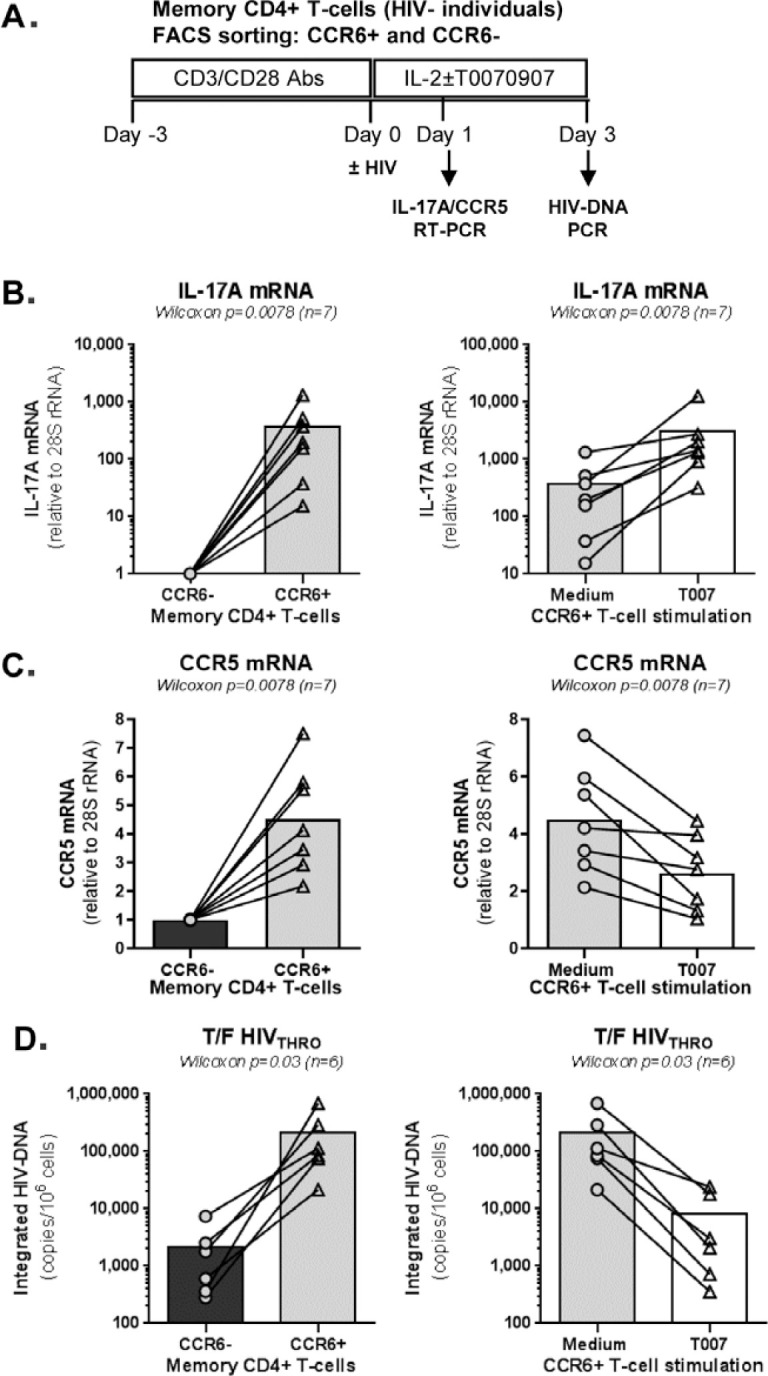
**T0070907 efficiently increases IL-17A expression and reduces HIV replication in sorted memory CCR6^+^ T cells. (A)** Shown is the experimental flow chart. Briefly, memory CCR6^+^ and CCR6^-^ T cells of HIV-uninfected individuals (n=6-7) were stimulated by CD3/CD28 for 3 days. **(B-C)** Cells were cultured in the presence of IL-2 and/or T0070907 (10μM) for 18 hours and RNA extraction was performed for RT-PCR quantification. Shown are results on **(B)** IL-17A (n=7) and **(C)** CCR5 (n=7) mRNA expression in CCR6^-^ versus CCR6^-^ T cells cultured in the absence of T0070907 (left panels) and CCR6^+^ T cells cultured in the presence/absence of T0070907 (right panels). Normalization was performed relative to 28S rRNA, with expression in CCR6-T cells being considered 1. **(D)** Another fraction of cells was exposed to T/F HIV_THRO_ strain (25 ng/10^6^ cells) and cultured in the presence of IL-2 and/or T0070907 (10μM) for 3 additional days. Shown are levels of HIV-DNA integration (as a measure of HIV replication) in CCR6^-^ versus CCR6^+^ T cells in the absence of T0070907 (left panel) and in CCR6^+^ T cells cultured in the presence/absence of T0070907 (right panel). The Wilcoxon signed rank test *P*-values are indicated on the graphs. Each symbol represents results generated with cells from one different donor; bars represent median values.

### PPARy antagonism boosts IL-17A expression and reduces HIV replication in CCR6^+^CD4^+^ T cells

IL-17A production and HIV permissiveness are key features of memory CCR6^+^CD4^+^ T-cells [14-16]. Thus, we further tested the immunological/virological effects of T0070907 in flow cytometry-sorted memory CCR6^+^ and CCR6^-^ T cells on HIV infection *in vitro* (Figure 4A). In the absence of T0070907, CCR6^+^ versus CCR6^-^ T cells expressed significantly higher levels of IL-17A and CCR5 mRNA (Figure 4B-C, left panels) and supported a more robust HIV-DNA integration (≈2 log_10_ difference) (Figure 4D, left panel). Similar to results on bulk memory T cells, T0070907 significantly increased IL-17A mRNA expression (Figure 4B, right panel) and reduced CCR5 mRNA expression as well as HIV-DNA integration in memory CCR6^+^ T cells (Figure 4C-D, right panels). Thus, consistent with superior expression of PPARy in CCR6^+^ Th17/Th1Th17-polarized versus CCR6^-^ Th1-polarized T cells [17, 18, 32, 34], T0070907 acted on CCR6^+^ T cells to upregu-late IL-17A production and limit HIV *de novo* infection by mechanisms including CCR5 down-regulation.

### RNA-Sequencing reveals a complex network of cellular processes positively or negatively regulated by PPARy in memory CCR6+CD4+ T cells

To get further insights into the mechanism of action of PPARy antagonism, genome-wide transcriptional profiling was performed in CCR6^+^ T cells stimulated via the TCR for 3 days and cultured in the presence or absence of T0070907 for an additional 18 hours (Figure 5A). Differentially expressed genes were classified based on *P* values (*P*) or adjusted *P* values (adj. *P*) and fold change (FC) gene expression. Profound transcriptional changes were induced by T0070907 in CCR6^+^ T cells, with 4,002 transcripts upregulated and 1,249 transcripts downregulated (adj. *P*<0.05; FC cutoff, 1.3) (Figure 5B), with the top 50 upregulated (adj. *P*<0.05; FC>8) and down-regulated (adj. *P*<0.05; FC<-3.2) transcripts listed in Supplemental Tables 1-2, respectively.

**Figure 5.**
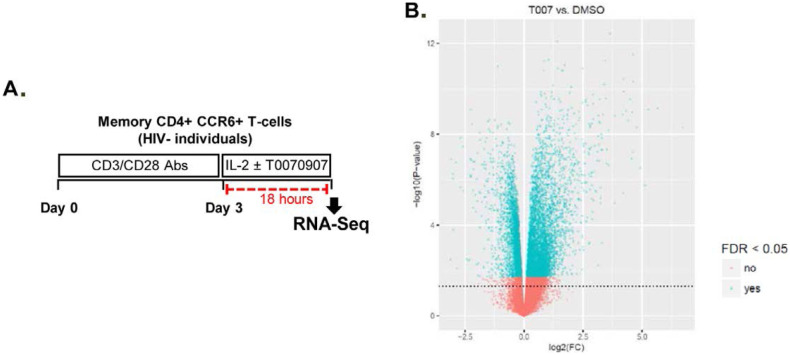
**T0070907 imprints CCR6^+^CD4^+^ T cells with an anti-viral transcriptional program. (A)** Shown is the experimental flow chart for genome-wide transcriptional analysis. Briefly, memory CCR6^+^ T cells of HIV-uninfected individuals (n=8) were stimulated by CD3/CD28 for 3 days and cultured with IL-2 in the presence/absence of T0070907 (10μM) for additional 18 hours. Total RNA was extracted for RNA sequencing. **(B)** Volcano plots for all probes in each linear model with the log_2_ FC on the x-axis and the negative logarithm of the adjusted *P*-values for false discovery rate (FDR) on the y-axis. The red/green color code is based on the 5% FDR threshold. **(C)** Heatmap represents 71 pathways included in the gene ontology (GO) classification: cytokines/chemokines (pink), drug transporters (blue), glucose/lipid metabolism (orange), and inflammation/immune response to type I interferon (violet) based on the 5% FDR threshold. Heatmap cells are scaled by the expression level z-scores for each probe individually. **(D)** Ingenuity pathway analysis (IPA) identified genes involved in HIV-1 production and differentially modulated by T0070907 (*P*<0.05). The y-axis represents the FC, with the 1.3 FC cut-off indicated by the dotted line. **(E)** IL-21 levels in cell culture supernatants were quantified by ELISA (n=5). Each symbol represents 1 different donor; bars represent median values. Wilcoxon matched-pairs signed rank test are indicated on the graphs.

**Supplementary Figure 5.**
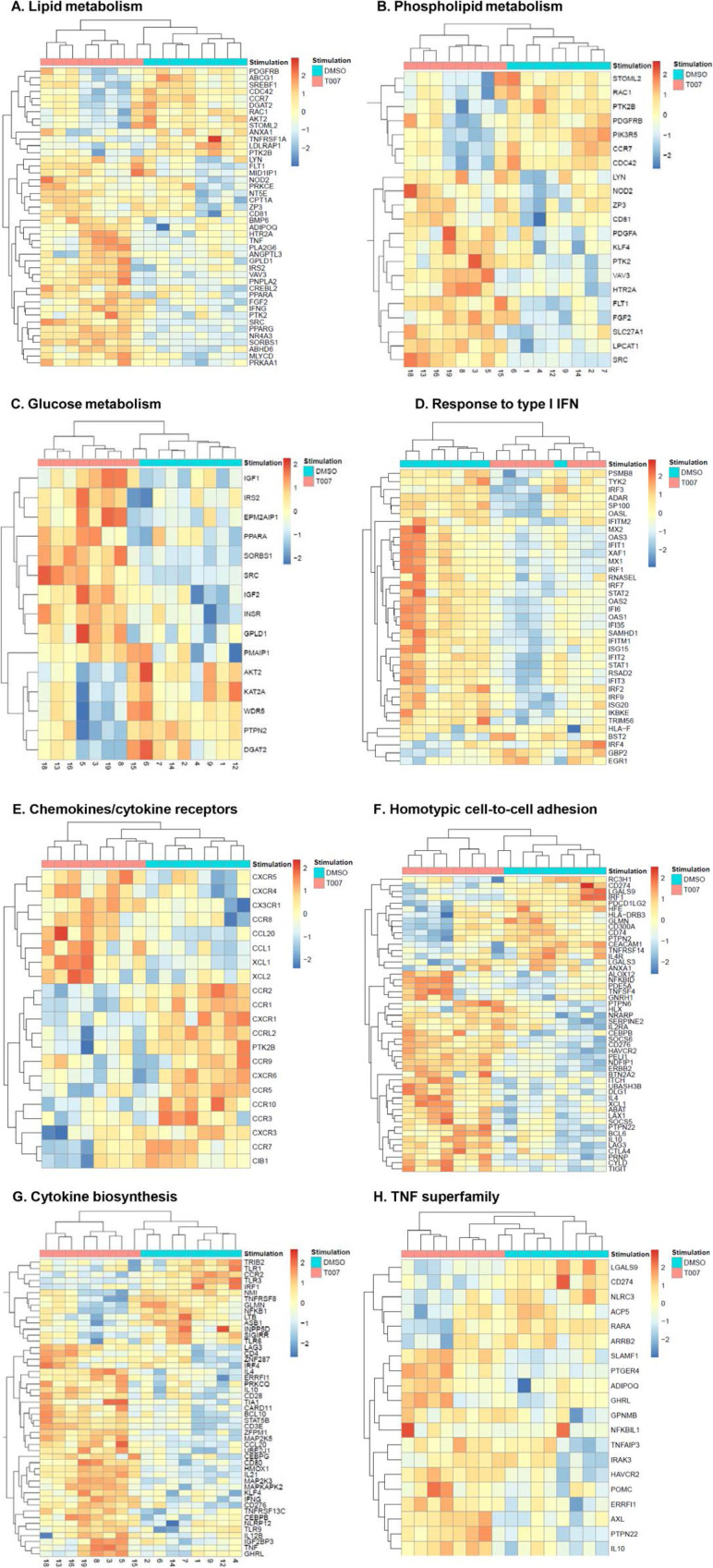
**Gene Ontology (GO) classification of transcripts modulated by T0070907 in CCR6+ T cells.** RNA-Seq transcriptional profiles were generated as described in Figure 5. Differentially expressed genes (*P*<0.05) were classified based on their biological functions using GO terms as follows: lipid metabolism (A), phospholipid metabolism (B), glucose metabolism (C), response to type I IFN (D), chemokines/cytokines receptors (E), homotypic cell-to-cell adhesion (F), cytokine biosynthesis (G), and TNF superfamily (H). Heatmap cells are scaled by the expression level z-scores for each probe individually. For each heatmap, gene expression values are represented as a gradient from red (highest expression) to blue (lowest expression). Results were generated with cells from n=8 donors, with each column representing 1 donor.

**Supplementary Figure 6.**
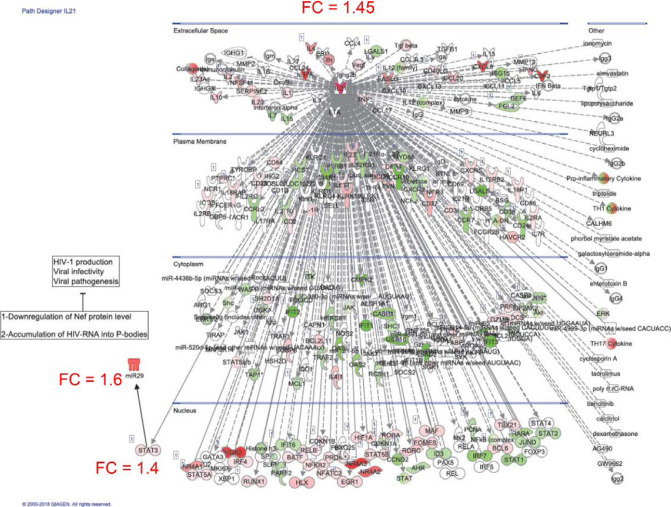
**Components of the IL-21 signalizing pathway modulated by T0070907 in CCR6+ T cells.** RNA-Seq transcriptional profiles were generated as described in Figure 5. Ingenuity pathway analysis (IPA) was used to illustrate gene networks associated with IL-21. The color code is based on the expression FC in T0070907-treated CCR6+ versus untreated CCR6+ T cells (red and green for upregulated and downregulated transcripts, respectively). FC expression relative to IL-21, STAT3, and miR-29 in T0070907-treated versus untreated CCR6+ T cells are illustrated. The miR-29 connection to the IL-21 network was added manually based on a recent publication demonstrating that IL-21 regulates its expression by STAT3 (75).

Gene Set Variation Analysis (GSVA) allowed the identification of Gene Ontology (GO) biological processes (false discovery rate (FDR) <0.05) using the Broad Institute data base (MSigDB C2, V6.2). Among 71 modulated pathways (Figure 5C), top pathways were linked to the GO terms: *i*) lipid/phospholipid and glucose metabolism (Supplemental Figure 6A-C), *ii*) inflammation/immune response to type I interferon (Supplemental Figure 5D), and *iii*) cytokines, chemokines and adhesion molecules (Supplemental Figure 5E-H). Differentially expressed genes linked to the GO term lipid/phospholipid metabolism, include the upregulation of the transcription factors PPARy, PPARα, KLF4, and NR4A3; the pattern recognition receptor NOD2; the tetraspanin CD81; the signaling molecules PTK2, PLA2G6, FGF2, and FLT1; the guanine nucleotide exchange factor VAV3; the hormone ADIPOQ/adiponectin; the cytokines TNF and IFNG; the downregulation of the ATP transporter ABCG1; the G protein RAC1; and the cell cycle regulator CDC42 (Supplemental Figure 5A-B). Differentially expressed genes linked to the GO term glucose metabolism include the upregulation of the glycosylphosphatidylinositol (GPI) degrading enzyme GPLD1, the insulin-like growth factors IGF1 and IGF2, and the phorbol-12-myristate-13-acetate-induced protein 1 (PMAIP1); and the downregulation of the enzymes tyrosine-protein phosphatase non-receptor type 2 (PTPN2) and diglyceride acyltransferase (DGAT2) (Supplemental Figure 5C). Differentially expressed genes linked to the GO term inflammation/immune response to type I interferon were mainly downregulated by T0070907 and included genes documented to play a positive/negative regulatory role in HIV replication such as ADAR, MX2, MX1, OAS1, RNASEL, SAMHD1, ISG15, ISG20, IFITM2, IFITM3, and TRIM56; of note, transcripts coding for the restriction factor BST2 were upregulated (Supplemental Figure 5D). Finally, Differentially expressed genes related to the GO terms cytokines, chemokines, and adhesion molecules included upregulated transcripts for chemokine receptors (CXCR5, CXCR4, CX3CR1, CCR8), chemokines (CCL20, CCL1, XCL1, XCL2), cell-to-cell adhesion molecules/immune checkpoints (CD276/B7-H3, LAG3, CTLA4, TIGIT), and cytokines/cytokine regulators (IL-4, IL-10, CD28, BCL10, STAT5B, CD3E, CD80, IL-21, KLF4, IFNG, TLR9, TNF, TNFAIP3, IRAK3, AXL, PTPN22); as well as downregulated transcripts for chemokine receptors (CCR1-3, CCR5, CCR7, CCR9, CCR10, CXCR3, CXCR6), chemokines (CCRL2), cell-to-cell adhesion molecules (CD274/PD-L1, LGALS9, CD300A, CD74, CEACAM1, TNFSF14, LGALS3, TNFSF4), and cytokine biosynthesis (TLR1, NFKB1, LTB, TLR6, NLRC3, RARA) (Supplemental Figure 5E-H).

**Figure 5C.**
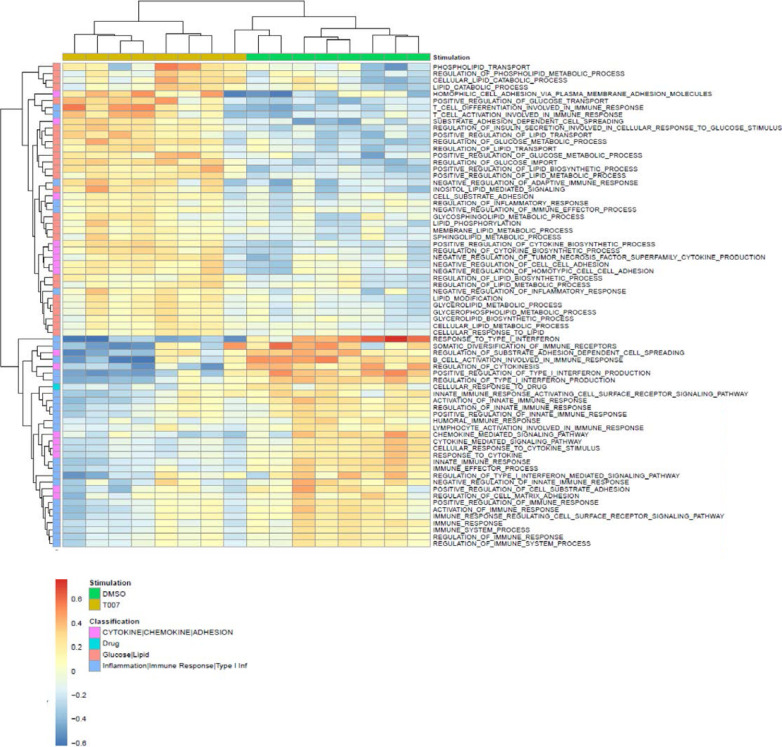
GSVA on GO pathways

**Figure 5D and 5E:**
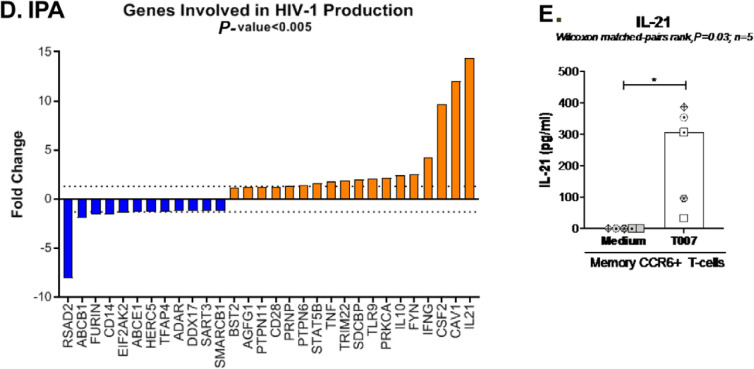


**Figure 6.**
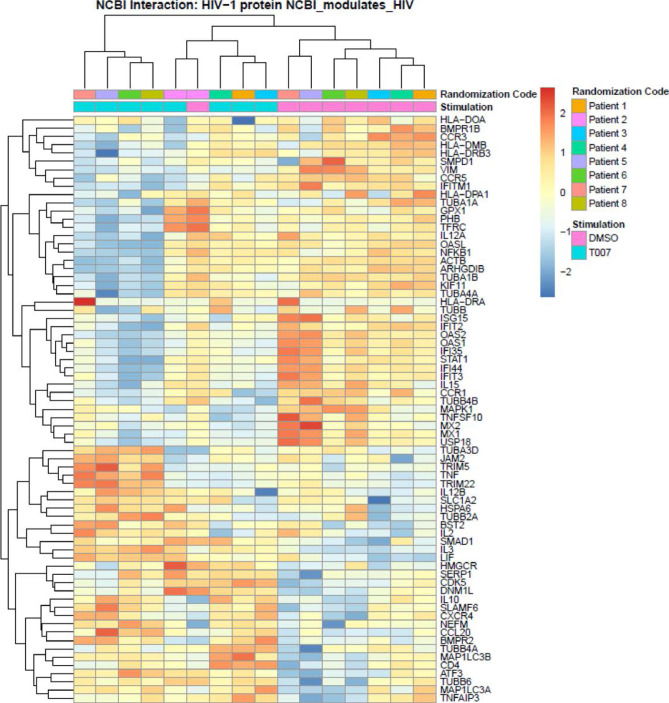
**Meta-analysis using the NCBI HIV interaction database.** Genome-wide transcriptional profiles were generated as in Figure 5. **(A)** Transcripts modulated by T0070907 in CCR6^+^ T cells (*P* <0.05, FC cutoff 1.3) were matched to the lists of human genes included on the NCBI HIV interaction database. Heat-map cells are scaled by the expression level z-scores for each probe individually. Results from each donor are indicated with a different color code (n=8).

These results reveal a previously unrecognized complex network of cellular processes that are positively/negatively controlled by PPARy in Th17-polarized CCR6^+^ T cells, with relevance for understanding the dichotomous effects of T0070907 on the various steps or HIV replication.

### A Tfh-specific transcriptional signature induced upon PPARy inhibition

Ingenuity Pathway Analysis revealed the upregulation and downregulation of transcripts previously linked to the negative (eg, IL-21, CAV1, BST2) and positive (eg, furin) regulation of HIV replication, respectively (Figure 5D). Considering the well-documented role of IL-21 in modulating Th17/Tfh survival [14, 15], as well as its antiviral properties [44, 45, 56], we pursued the validation of IL-21 at the protein level. Results generated with memory CCR6^+^ T cells from 5 individuals confirmed the significant upregulation of IL-21 protein production by T0070907 (Figure 5E). IL-21 exerts its antiviral functions by the induction of miR-29 [44], a non-coding RNA that reduces HIV replication by interfering with Nef [57]. Consistently, Ingenuity Pathway Analysis (Supplemental Figure 6) revealed the interactome linked to IL-21 and the connection with miR-29 by the up-regulation of STAT3 [44]. These results point to IL-21 upregulation as one mechanism underlining the virological features of PPARy inhibition.

**Supplementary Figure 7.**
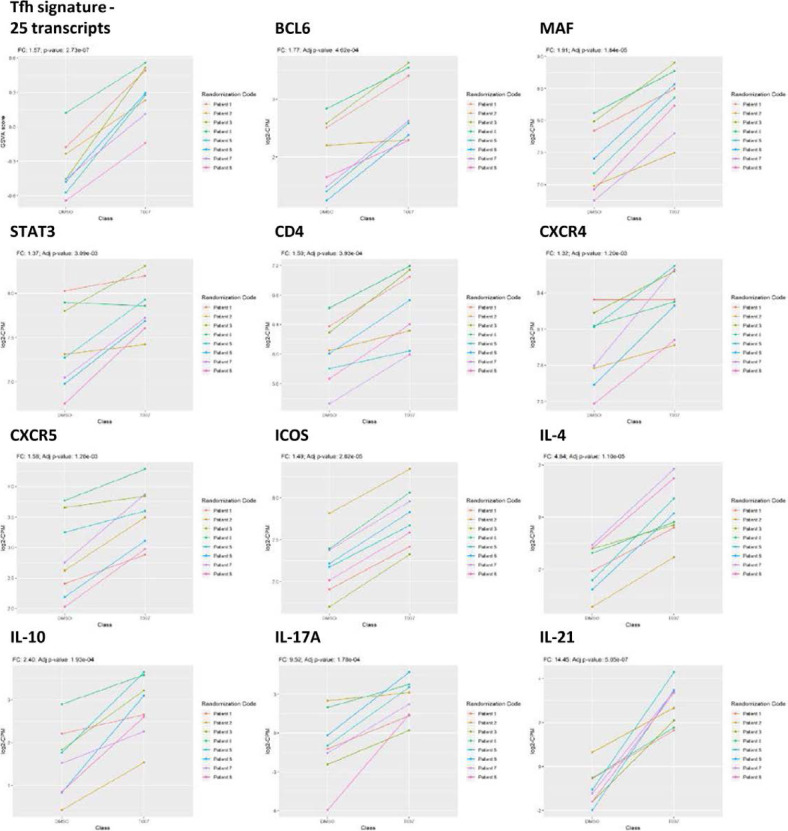
**T0070907 acts on CCR6+ T cells to promote expression of follicular helper T-cell (Tfh) marker transcripts.** RNA-Seq transcriptional profiles were generated as described in Figure 5. An intelligent search was performed to identify Tfh-specific transcripts among transcripts upregulated by T0070907 in CCR6+ T cells. Shown are changes in the expression of 25 Tfh-specific transcripts identified in the literature (Crotty, 2019), as well as individual Tfh-specific transcription factors (BCL6, MAF, and STAT3), surface molecules (CD4, CXCR4, CXCR5, and ICOS), and cytokines (IL-4, IL-10, IL-17A, and IL-21). Results from n=8 individual donors are represented in different colors.

In addition to IL-21, T0070907 upregulated a set of Tfh-specific transcripts [58], including transcription factors (Bcl6, MAF, STAT3), chemokine receptors (CXCR4, CXCR5), surface markers (CD4, ICOS), and cytokines (IL-4, IL-10, IL-17A/F) (Supplemental Figure 7).

**Figure 7.**
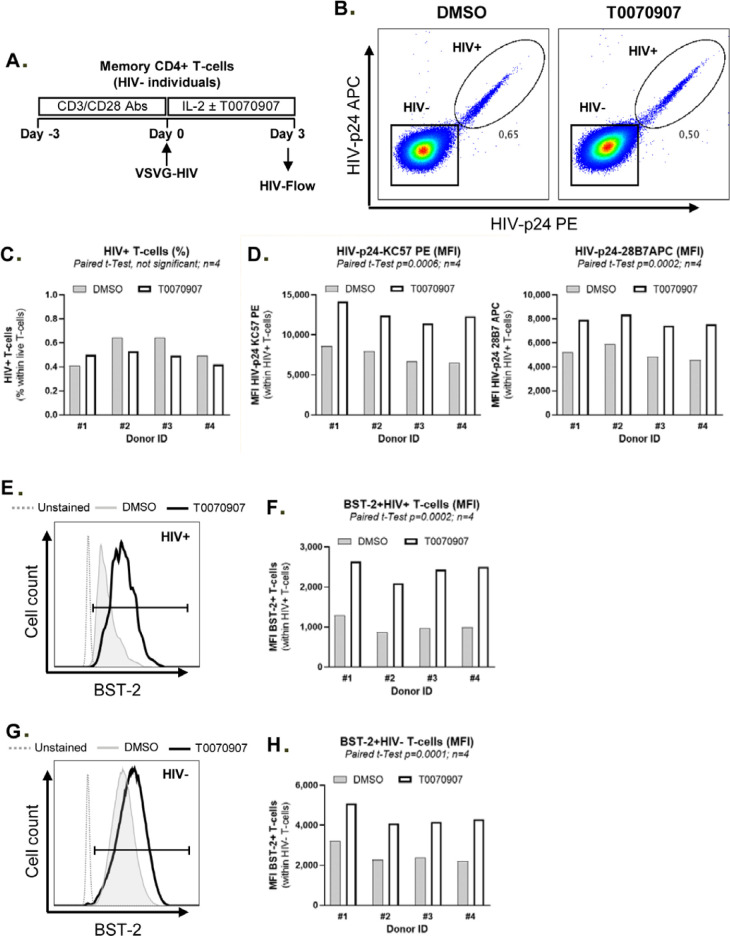
**T0070907 prevents BST-2 downregulation on HIV-infected T cells. (A)** Shown is the experimental flow chart. Briefly, memory CD4^+^ T cells isolated from HIV-uninfected individuals were stimulated with anti-CD3/CD28 antibodies for 3 days, exposed to single round VSV-G/HIV for 3 hours. Then, cells were cultured in the presence of IL-2 (5 ng/ml) and in the presence or the absence (DMSO) of T0070709 (10μM) for 3 additional days. HIV-Flow using 2 distinct HIV-p24 antibody clones coupled with different fluorochromes (28B7 APC and PE), together with surface staining with BST-2 and CD4 antibodies, were performed and analyzed by flow cytometry. Shown is the co-expression of HIV-p24 PE and APC antibodies allowing the identification of productively infected cells (HIV^+^) in 1 representative donor **(B)** and the statistical analysis of the % of HIV^+^ cells **(C)** and the MFI of HIV-p24 PE and APC expression on exposure to DMSO or T0070907 in 4 different donors **(D)**. Shown are histograms from 1 representative donor for BST-2 and CD4 expression **(E and G),** as well as the statistical analyses of BST-2 and CD4 expression (% and MFI) on HIV^+^ cells in 4 different donors **(F and H)**. Paired *t*-test values are indicated on the graphs.

### HIV-dependency factors modulated by PPARy inhibition

**Supplementary Figure 8.**
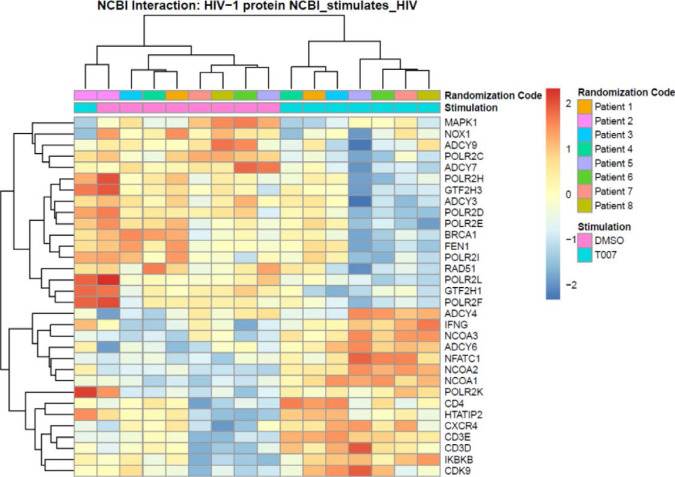
**Meta-analysis using the NCBI HIV interaction database.** RNA-Seq transcriptional profiles were generated as described in Figure 5. Transcripts differentially expressed in CCR6+ T cells cultured in the presence/absence of T0070907 (*P*< 0.05, FC cut-off 1.3) were matched to the lists of human genes previously identified to interact with HIV-1 proteins (NCBI Interactor database). Heatmap cells are scaled by the expression level z-scores for each probe individually. For each heatmap, gene expression values are represented as a gradient from red (highest expression) to blue (lowest expression). Results from each donor are indicated with a different color code (n=8).

A meta-analysis using the NCBI HIV-1 interactions database allowed the identification of human genes previously involved in HIV-1 infection that are modulated by T0070907 in CCR6^+^ T cells. Specifically, TRIM5, TNF, TRIM22, BST2, IL-2, IL-3, LIF, IL-10, CXCR4, SERP1, and CD4 were upregulated; while VIM, CCR5, IFITM1, OASL, NFKB1, ISG15, IFIT2, OAS2, OAS1, IFIT35, STAT1, IL15, MX2, MX1, and USP18 were downregulated (Figure 6). Among transcripts regulating HIV transcription, T0070907 upregulated the expression of the nuclear receptor co-activators (NCOA)1-3, the nuclear factor of activated T cells cytoplasmic 1 (NFATC1), the HIV-1 Tat Interactive Protein 2 (HTATIP2), CD3E, CD3D, IKBKB, and CDK9, and it downregulated the expression of MAPK1, NOX1, and the DNA-directed RNA polymerases POLR2C, POLR2H, POLR2D, POLR2E, POLR2F, and POLR2L (Supplemental Figure 8).

**Figure 8.**
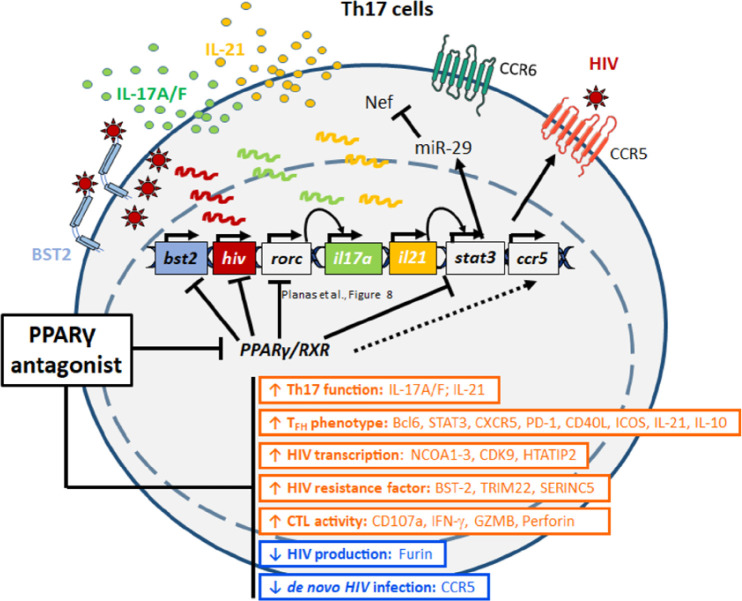
**Summary of PPARy antagonism-mediated virological/immunological reprogramming of CCR6^+^ T cells.** In line with the documented capacity of PPARy to repress HIV and RORyt transcription, the PPARy antagonist T0070907 acted on CCR6^+^ Th17 cells to boost both HIV transcription (NCOA1-3, HTATIP2, CDK9) and the expression of specific Th17/Tfh transcripts (eg, IL-17A, IL-21). Unexpectedly, the PPARy antagonism prevented *de novo* production/release of virions from reservoir cells by negatively interfering with multiple steps of the HIV replication cycle, from virion maturation (eg, furin) and viral particle release (eg, BST2), to viral entry into new target cells (eg, CCR5), as well as the IL-21/miR-29 antiviral axis. Thus, the PPARy antagonism may represent a new strategy to eradicate HIV reservoirs in Th17 cells. **Table 1**: Clinical parameters of ART-treated PLWH study participants.

Together, these RNA-Seq results reveal that T0070907-mediated transcriptional reprogramming is associated with the negative regulation of multiple steps of the viral replication cycle such as CCR5-mediated entry, the uncoating (eg, TRIM5), reverse transcription (eg, SAMHD1), Nef-mediated functions (eg, IL-21, miR29), viral particle production (eg, TRIM22), release (eg, BST2), Env processing (eg, furin), while facilitating HIV transcription (eg, NCOA1-3, HTATIP2, CDK9), and Th17-specific effector functions (eg, RORyt, STAT3, IL-17A, IL-21).

### PPARy inhibition prevents BST-2 downregulation on HIV-infected CD4^+^ T cells

**Supplementary Figure 9.**
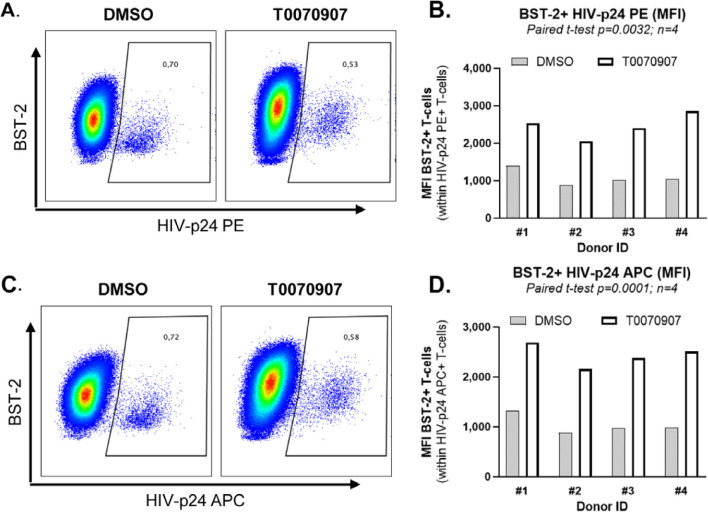
**T0070907 prevents BST-2 downregulation on HIV-infected cells.** Experiments were performed as described in Figure 7 legend. Shown are dot plots of BST-2 and HIVp-24 PE or HIV-p24 APC co-expression (A and C), as well as the statistical analyses for BST-2 expression (MFI) on HIV-p24 PE+ and HIV-p24 APC+ T different donors (B and D). Paired t-test values are indicated on the graphs.

Tetherin/BST2 represents a key HIV restriction factor downregulated by the HIV accessory protein Vpu to allow release of progeny virions from productively infected T cells [59-61]. Our RNASeq revealed the upregulation of BST-2 RNA on memory CCR6^+^ T cells on exposure to T0070907 (FC: 1.2, *P*=0.001; adj. *P*=0.007) (Figure 6, Supplemental Figures 5D and 8). The interrogation of the ENCODE dataset generated by TF ChIP-Seq analysis on HepG2 cells (https://www.encodeproject.org/experiments/ENCSR130VQL/) allowed us to identify 2,118 T0070907-modulated transcripts that are putative direct PPARy targets in T cells, with BST-2 encoding for PPREs in its promoter (data not shown). Thus, we hypothesized that increased BST-2 expression contributes to limiting release of progeny virions from infected cells, as demonstrated in Figure 1F. To test this hypothesis, we performed single-round HIV infection using a VSV-G/HIV construct (which enters cells by endocytosis independently of CD4 and co-receptors [49]), cultured cells in the presence or absence of T0070907, and analyzed by FACS the expression of BST-2 protein on the surface of HIV-infected cells identified using HIV-Flow (Figure 7A), as previously reported [62]. As expected, in the absence of T0070907, BST-2 expression was downregulated on HIV-infected compared to uninfected bystander T cells (Supplemental Figure 9). Exposure to T0070907 led to a significant increase in the intracellular HIV-p24 expression (MFI of HIV-p24 PE and HIV-p24 APC antibody expression) (Figure 7B-D), as well as an increased BST-2 surface expression (MFI) on HIV-infected T cells (Figure 7E-F, Supplemental Figure 9). A T0070907-mediated increase in BST-2 expression was also observed on the surface of bystander HIV-uninfected T cells (Figure 7G-H, Supplemental Figure 9). These results indicate that PPARy inhibition allows efficient HIV translation into proteins (ie, HIV-p24) and suggest that BST-2 upregulation by T00709070 contributes to limiting the release of progeny virions from productively infected T cells.

## DISCUSSION

In this study, we reveal the unique features combined by the PPARy antagonist T0070907 including the positive regulation of HIV transcription/translation and Th17/Tfh-specific effector functions in memory CD4^+^ T cells of ART-treated PLWH, together with its capacity to reduce *de novo* virion production and/or spread from HIV reservoir cells. By using a genome-wide transcriptional profiling in Th17-polarized CCR6^+^CD4^+^ T cells, we revealed a complex transcriptional reprogramming underlying the observed immunological/virological features of T0070907, with antiviral mechanisms located at multiple steps of the HIV replication cycle downstream translation, including the BST-2-mediated restriction of HIV release (Figure 8).

In addition to the knowledge that PPARy acts as a repressor of HIV [31] and RORyt transcription [32, 34], we demonstrate that the pharmacological inhibition of PPARy using the antagonist T0070907 [53] boosted HIV transcription and RORyt-mediated transcription of Th17-specific genes. Conversely, we observed an unexpected block in the viral production and release and/or spread in culture observed during viral outgrowth *ex vivo* and HIV infection *in vitro*. Of particular importance, T0070907 acted preferentially on CCR6^+^ Th17-polarized T cells, a subset known to be enriched in HIV reservoirs in ART-treated PLWH [16, 63], to increase IL-17A production and reduce CCR5 expression and viral replication *in vitro*. Similar to T0070907, the literature documents that PKC-θ activators such as prostratin, a non-tumor-promoting phorbol ester, also acts as an LRA while blocking *de novo* HIV production to mediate the elimination of HIV reservoirs by a kick and kill strategy [64-67]. Whether the effects of prostratin and its derivatives [68] also involve PPARy-modulated processes remains to be determined. However, one major difference is that PKC-θ activators downregulate CD4, while T0070907 does not.

The PPARy/RXR heterodimer is known to target genes involved in lipid metabolism such as cholesterol and fatty acids that influence multiple aspects of antiviral immunity [26, 28, 69]. Among oxysterols presenting antiviral properties, 25HC, metabolized from cholesterol by the enzyme CH25H, blocks the replication of HIV by acting on the viral entry but not transcription [70], with the effects on the post-transcriptional steps of the replication cycle remaining unexplored. In addition, 25HC has been identified as a natural ligand for RORyt [71, 72]. The fact that PPARy deficiency was linked to CH25H overexpression [73], prompted our initial hypothesis that T0070907 blocks HIV outgrowth *ex vivo* and infection *in vitro* and boosts Th17 effector functions via CH25H/25HC-dependent mechanisms. In agreement, T0070907 upregulated the expression of CH25H mRNA in TCR-activated CCR6^-^ non-Th17 cells (data not shown), further explaining their relative resistance to HIV infection [14, 15]. However, CH25H mRNA was undetectable in CCR6^+^ Th17 cells (data not shown), indicating that T0070907 exerts its antiviral effects in Th17 cells via CH25H/25HC-independent mechanisms.

To investigate mechanisms by which T0070907 disconnects HIV transcription from downstream viral replication steps, we performed a genome-wide transcriptional profiling using the RNA-Seq Illumina technology. GSVA identified activation of pathways linked to lipid/phospholipid and glucose metabolism. Metabolic reprogramming during TCR triggering trains T cells to integrate immunological and metabolic information required for the subsequent acquisition of specific effector functions [74]. Glucose metabolism has been identified to play a central role in HIV replication, with the glucose transporter GLUT1 being a marker for HIV permissive T cells [75]. Metabolism disruption is associated with HIV disease progression, with higher glucose uptake being observed in CD4^+^ T cells of PLWH compared to non-infected individuals [76]. Recent studies linked the susceptibility to HIV infection to the metabolic status of specific CD4^+^ T-cell subsets [77]. Changes in the CD4^+^ T-cell metabolic program are controlled by the mTORC1/PPARy axis [74, 78]. In line with this, T0070907 upregulated genes associated with PI3K/Akt signaling, a pathway known to promote mTOR activation [15]. Indeed, several groups including ours, identified mTOR as a positive regulator of HIV replication [20], acting at the level of viral entry [79] and transcription [80, 81]. Indeed, in preliminary studies, we demonstrated that TCR triggering in the presence of T0070907 leads to increased mTOR phosphorylation. Therefore, the activation of the PI3K/Akt pathway in the presence of T0070907 might be in part responsible of the increase in HIV transcription, likely via mTOR-dependent mechanisms.

GSVA identified pathways modulated by T0070907 in CCR6^+^ T cells revealed that PPARy antagonism produces profound transcriptional modifications linked to the metabolism of cellular membrane components, including glycosaminoglycan, glycosphingolipid, and sphingolipid. These components of the cellular membrane play a key role in membrane organization and membrane raft formations [29]. Membrane receptors such as the HIV co-receptors CCR5/CXCR4 are recruited to the membrane raft, and the clustering of these receptors promotes HIV entry into target cells [82]. In addition, membrane rafts play a crucial role in HIV-1 assembly and release [83, 84]. Therefore, modification of the cellular composition and membrane raft formation by T0070907 may contribute to the decreased HIV entry/release; additional investigations are needed to clarify this. The formation of biofilms rich in collagen and cell-host molecules such as tetherin/BST2 has been reported for human T-cell leukemia virus type 1 (HTLV-1) [85]. The possibility that other viruses such as HIV form biofilms remains to be determined [86]. Of note, the main upregulated gene by T0070907 is fibromodulin (FMOD), a component of the extracellular matrix which participates in the assembly of collagen fibers. In line with this, the collagen triple helix repeat containing 1 (CTHRC1) and the tetherin/BST2 transcripts were upregulated by T0070907. These findings indicate that T0070907 facilitates the establishment of biofilms able to trap newly produced virions thus preventing their spreading.

The GSVA of GO pathways also revealed the downregulation of pathways/transcripts linked to interferon responses. Multiple interferon-stimulated genes (ISG), documented to restrict HIV replication, were downregulated by T0070907 in CCR6^+^ T cells. Among these transcripts, we noted a decreased expression of SAMHD1, which limits HIV reverse transcription and promotes HIV-RNA degradation [87]; MX2, which limits viral decapsidation, pre-integration complex formation and nuclear import [88, 89]; IFITM2 and IFITM3, known to interact with HIV-1 Env in infected cells and impair Env processing and incorporation into virions [90]; and ISG15, known to induce ISGylation of viral Gag proteins and impeded HIV release [91]. These results point to a previously unrecognized implication of PPARy in the positive transcriptional regulation of specific HIV-restriction factors, including SAMHD1, MX2, IFITM2, IFITM3, BST2, and ISG15, in line with the antiviral program promoted by PPARy activation [17].

Our RNA-Seq results also revealed a T0070907-mediated increase in the expression of the classical Tfh markers CXCR5, ICOS, BCL6, PD-1, CD40L, IL-10, and IL-21. In line with this, previous studies demonstrated that PPARy activation prevents Tfh differentiation [33]. Of note, by boosting IL-21 production T0070907 may improve Th17/Tfh survival and their effector functions. Indeed, in a model of SIV infection, the IL-21 supplementation of ART reduced inflammation, restored mucosal Th17 frequency, decreased the size of viral reservoir [45, 46], and also delayed viral rebound on ART interruption [45]. In addition, IL-21 exhibited antiviral functions by the induction of miR-29 [44] that targeted HIV-Nef for degradation [57] S. K.</author></authors></contributors><auth-address>Institute of Genomics and Integrative Biology (IGIB. The IL-21/miR-29 axis was also linked to slowing of HIV disease progression [56]. Therefore, the IL-21/miR-29 axis is highly likely to contribute to the antiviral effects of PPARy antagonism.

The meta-analysis performed using the NCBI HIV-1 interaction database pointed to additional T0070907-mediated antiviral mechanisms. Specifically, T0070907 upregulated expression of CAV1, reported to inhibit HIV particle production in macrophages [92]; SERINC5, which is incorporated into virions and prevents the fusion of the virion with the cellular membrane of a new target cell [93]; TRIM22, which blocks Gag migration to the plasma membrane and inhibits HIV particle production [94]; and BST2, which limits viral particle release [87]. A T0070907-mediated upregulation of the HIV restriction factor TRIM5α, which interacts with the HIV capsid and induces its proteasomal degradation leading to premature decapsidation [95], was also observed. Finally, T0070907 downregulated furin, a protease preferentially expressed in Th17 cells [17, 18] and involved in HIV protein Env maturation and virion infectivity [96]. Thus, the antiviral features of T0070907 involve mechanisms dependent on CAV1, SERINC5, TRIM22, and BST2 over-expression, as well as furin downregulation, thus explaining a post-transcriptional block in HIV virion production and/or release.

Finally, the counterintuitive capacity of PPARy antagonism to decrease viral release/outgrowth while increasing viral transcription prompted us to focus on Tetherin/BST-2, an HIV restriction factor counteracted by Vpu and documented to mediate HIV tethering on the surface of infected cells [59-61]. Of note, T0070907 increased BST-2 mRNA expression in uninfected CCR6^+^CD4^+^ T cells. In a model of single round VSV-G/HIV infection *in vitro*, as expected, BST-2 protein expression was downregulated on infected T cells in the absence of T0070907. In contrast, the BST-2 expression was significantly higher on the surface of infected cells exposed to T0070907. An *in silico* search using the ENCODE database revealed that BST-2 encodes PPREs in its promoter and represents a putative direct PPARy target in CD4^+^ T cells. Thus, PPARy inhibition boosts HIV reactivation, while preventing progeny virion release from infected cells via BST-2-dependent mechanisms. The recognition of such reactivated viral reservoirs by antibodies and immune cells for subsequent clearance will be key for HIV cure. Future studies *in vitro* and in preclinical models are needed to determine whether PPARy antagonism promotes HIV reservoir purging in shock and kill strategies.

In conclusion, our results reveal complex previously unrecognized PPARy-dependent host-cell molecular circuits involved in the positive, as well as the negative regulation of various steps of the HIV replication cycle and demonstrate the possibility of disconnecting HIV transcription and translation from viral particle production/release (Figure 8). The efficacy of the PPARy antagonism in boosting IL-21 production is of major importance, considering IL-21 paucity during HIV infection [14, 15] and its documented antiviral/immune-regulatory features [44-46, 56]. Therefore, the pharmacological inhibition of PPARy may represent a new promising therapeutic strategy to boost Th17-effector functions that are key for mucosal immunity restoration and to promote HIV-reservoir purging in ART-treated PLWH.

## Figures and Tables

**Supplementary Table 1:** Transcripts up-regulated by T0070907 in memory CCR6+ T-cells

Gene ID	FC	Adj. P	Gene symbol	Gene description
ENSG00000122176	105,9	9,47E-07	FMOD	fibromodulin [Source:HGNC Symbol;Acc:HGNC:3774]
ENSG00000253958	50,6	3,44E-07	CLDN23	claudin 23 [Source:HGNC Symbol;Acc:HGNC:17591]
ENSG00000167157	38,5	3,60E-07	PRRX2	paired related homeobox 2 [Source:HGNC Symbol;Acc:HGNC:21338]
ENSG00000117281	34,8	4,89E-05	CD160	CD160 molecule [Source:HGNC Symbol;Acc:HGNC:17013]
ENSG00000173404	30,4w	1,42E-05	INSM1	INSM transcriptional repressor 1 [Source:HGNC Symbol;Acc:HGNC:6090]
ENSG00000109099	27,3	9,46E-07	PMP22	peripheral myelin protein 22 [Source:HGNC Symbol;Acc:HGNC:9118]
ENSG00000164932	24,5	6,72E-08	CTHRC1	collagen triple helix repeat containing 1 [Source:HGNC Symbol;Acc:HGNC:18831]
ENSG00000178947	24,5	2,05E-08	SMIM10L2A	small integral membrane protein 10 like 2A [Source:HGNC Symbol;Acc:HGNC:34499]
ENSG00000171724	24,4	5,02E-06	VAT1L	vesicle amine transport 1 like [Source:HGNC Symbol;Acc:HGNC:29315]
ENSG00000131831	23,9	4,20E-07	RAI2	retinoic acid induced 2 [Source:HGNC Symbol;Acc:HGNC:9835]
ENSG00000165895	23,6	7,73E-06	ARHGAP42	Rho GTPase activating protein 42 [Source:HGNC Symbol;Acc:HGNC:26545]
ENSG00000198535	22,6	1,84E-07	C2CD4A	C2 calcium dependent domain containing 4A [Source:HGNC Symbol;Acc:HGNC:33627]
ENSG00000164399	21,3	6,57E-07	IL3	interleukin 3 [Source:HGNC Symbol;Acc:HGNC:6011]
ENSG00000135960	20,1	3,78E-07	EDAR	ectodysplasin A receptor [Source:HGNC Symbol;Acc:HGNC:2895]
ENSG00000119508	18,6	1,41E-07	NR4A3	nuclear receptor subfamily 4 group A member 3 [Source:HGNC Symbol;Acc:HGNC:7982]
ENSG00000164684	18,6	5,54E-08	ZNF704	zinc finger protein 704 [Source:HGNC Symbol;Acc:HGNC:32291]
ENSG00000259422	18,4	8,48E-05		
ENSG00000255693	18,2	4,82E-07	LINC02389	long intergenic non-protein coding RNA 2389 [Source:HGNC Symbol;Acc:HGNC:53316]
ENSG00000138684	14,5	5,05E-07	IL21	interleukin 21 [Source:HGNC Symbol;Acc:HGNC:6005]
ENSG00000244457	14,3	1,78E-04	ENO1P1	enolase 1 pseudogene 1 [Source:HGNC Symbol;Acc:HGNC:3352]
ENSG00000150687	12,5	7,55E-09	PRSS23	serine protease 23 [Source:HGNC Symbol;Acc:HGNC:14370]
ENSG00000105974	12,1	1,18E-05	CAV1	caveolin 1 [Source:HGNC Symbol;Acc:HGNC:1527]
ENSG00000236320	12,0	1,11E-06	SLFN14	schlafen family member 14 [Source:HGNC Symbol;Acc:HGNC:32689]
ENSG00000248473	11,4	1,04E-05	LINC01962	long intergenic non-protein coding RNA 1962 [Source:HGNC Symbol;Acc:HGNC:52787]
ENSG00000106236	11,4	4,27E-06	NPTX2	neuronal pentraxin 2 [Source:HGNC Symbol;Acc:HGNC:7953]
ENSG00000122877	11,1	1,84E-07	EGR2	early growth response 2 [Source:HGNC Symbol;Acc:HGNC:3239]
ENSG00000080573	10,8	2,05E-08	COL5A3	collagen type V alpha 3 chain [Source:HGNC Symbol;Acc:HGNC:14864]
ENSG00000253304	10,5	8,21E-08	TMEM200B	transmembrane protein 200B [Source:HGNC Symbol;Acc:HGNC:33785]
ENSG00000205795	10,2	4,82E-07	CYS1	cystin 1 [Source:HGNC Symbol;Acc:HGNC:18525]
ENSG00000205502	9,9	2,01E-06	C2CD4B	C2 calcium dependent domain containing 4B [Source:HGNC Symbol;Acc:HGNC:33628]
ENSG00000235304	9,9	4,31E-06	LINC01281	long intergenic non-protein coding RNA 1281 [Source:HGNC Symbol;Acc:HGNC:50337]
ENSG00000196302	9,8	3,54E-03		
ENSG00000255026	9,8	4,31E-06		
ENSG00000164400	9,7	1,53E-06	CSF2	colony stimulating factor 2 [Source:HGNC Symbol;Acc:HGNC:2434]
ENSG00000112115	9,5	1,78E-04	IL17A	interleukin 17A [Source:HGNC Symbol;Acc:HGNC:5981]
ENSG00000106025	9,5	5,82E-05	TSPAN12	tetraspanin 12 [Source:HGNC Symbol;Acc:HGNC:21641]
ENSG00000204671	9,5	1,21E-04	IL31	interleukin 31 [Source:HGNC Symbol;Acc:HGNC:19372]
ENSG00000157680	9,3	7,61E-07	DGKI	diacylglycerol kinase iota [Source:HGNC Symbol;Acc:HGNC:2855]
ENSG00000105928	9,2	1,84E-07	GSDME	gasdermin E [Source:HGNC Symbol;Acc:HGNC:2810]
ENSG00000120549	9,1	7,24E-07	KIAA1217	KIAA1217 [Source:HGNC Symbol;Acc:HGNC:25428]
ENSG00000173210	9,1	8,78E-06	ABLIM3	actin binding LIM protein family member 3 [Source:HGNC Symbol;Acc:HGNC:29132]
ENSG00000153208	9,0	3,19E-06	MERTK	MER proto-oncogene, tyrosine kinase [Source:HGNC Symbol;Acc:HGNC:7027]
ENSG00000198734	8,9	5,05E-07	F5	coagulation factor V [Source:HGNC Symbol;Acc:HGNC:3542]
ENSG00000082781	8,9	1,76E-06	ITGB5	integrin subunit beta 5 [Source:HGNC Symbol;Acc:HGNC:6160]
ENSG00000262526	8,8	1,97E-02		
ENSG00000156535	8,7	5,02E-06	CD109	CD109 molecule [Source:HGNC Symbol;Acc:HGNC:21685]
ENSG00000134489	8,5	8,60E-06	HRH4	histamine receptor H4 [Source:HGNC Symbol;Acc:HGNC:17383]
ENSG00000169429	8,4	7,78E-05	CXCL8	C-X-C motif chemokine ligand 8 [Source:HGNC Symbol;Acc:HGNC:6025]
ENSG00000171658	8,1	7,17E-05	NMRAL2P	NmrA like redox sensor 2, pseudogene [Source:HGNC Symbol;Acc:HGNC:52332]
ENSG00000087494	8,0	3,39E-06	PTHLH	parathyroid hormone like hormone [Source:HGNC Symbol;Acc:HGNC:9607]
ENSG00000272273	7,8	1,88E-05	IER3-AS1	IER3 antisense RNA 1 [Source:HGNC Symbol;Acc:HGNC:53629]
ENSG00000141574	7,7	6,22E-07	SECTM1	secreted and transmembrane 1 [Source:HGNC Symbol;Acc:HGNC:10707]
ENSG00000250295	7,7	1,49E-04	RDH10-AS1	RDH10 antisense RNA 1 [Source:HGNC Symbol;Acc:HGNC:51658]
ENSG00000143878	7,6	2,41E-07	RHOB	ras homolog family member B [Source:HGNC Symbol;Acc:HGNC:668]
ENSG00000100292	7,5	1,52E-07	HMOX1	heme oxygenase 1 [Source:HGNC Symbol;Acc:HGNC:5013]
ENSG00000170647	7,5	3,46E-04	-	-
ENSG00000101134	7,4	5,93E-04	DOK5	docking protein 5 [Source:HGNC Symbol;Acc:HGNC:16173]
ENSG00000074410	7,3	4,89E-05	CA12	carbonic anhydrase 12 [Source:HGNC Symbol;Acc:HGNC:1371]
ENSG00000143184	7,3	1,91E-05	XCL1	X-C motif chemokine ligand 1 [Source:HGNC Symbol;Acc:HGNC:10645]
ENSG00000165457	7,3	1,32E-04	FOLR2	folate receptor beta [Source:HGNC Symbol;Acc:HGNC:3793]
ENSG00000259363	7,3	7,78E-07		
ENSG00000110675	7,2	2,06E-05	ELMOD1	ELMO domain containing 1 [Source:HGNC Symbol;Acc:HGNC:25334]
ENSG00000088826	7,2	3,65E-06	SMOX	spermine oxidase [Source:HGNC Symbol;Acc:HGNC:15862]
ENSG00000225125	7,1	2,57E-05	RANP4	RAN, member RAS oncogene family pseudogene 4 [Acc:HGNC:39859]
ENSG00000236833	7,0	8,82E-07		
ENSG00000235488	7,0	6,69E-05	JARID2-AS1	JARID2 antisense RNA 1 [Source:HGNC Symbol;Acc:HGNC:40314]
ENSG00000124212	6,9	9,46E-07	PTGIS	prostaglandin I2 synthase [Source:HGNC Symbol;Acc:HGNC:9603]
ENSG00000270164	6,9	4,25E-06	LINC01480	long intergenic non-protein coding RNA 1480 [Source:HGNC Symbol;Acc:HGNC:51124]
ENSG00000132329	6,8	8,12E-07	RAMP1	receptor activity modifying protein 1 [Source:HGNC Symbol;Acc:HGNC:9843]
ENSG00000116981	6,8	3,47E-03	NT5C1A	5'-nucleotidase, cytosolic IA [Source:HGNC Symbol;Acc:HGNC:17819]
ENSG00000108702	6,8	3,83E-03	CCL1	C-C motif chemokine ligand 1 [Source:HGNC Symbol;Acc:HGNC:10609]
ENSG00000243244	6,8	4,07E-06	STON1	stonin 1 [Source:HGNC Symbol;Acc:HGNC:17003]
ENSG00000124466	6,8	2,23E-05	LYPD3	LY6/PLAUR domain containing 3 [Source:HGNC Symbol;Acc:HGNC:24880]
ENSG00000163053	6,7	5,71E-06	SLC16A14	solute carrier family 16 member 14 [Source:HGNC Symbol;Acc:HGNC:26417]
ENSG00000153234	6,7	5,27E-06	NR4A2	nuclear receptor subfamily 4 group A member 2 [Source:HGNC Symbol;Acc:HGNC:7981]
ENSG00000121039	6,5	2,68E-07	RDH10	retinol dehydrogenase 10 [Source:HGNC Symbol;Acc:HGNC:19975]
ENSG00000132465	6,4	5,19E-04	JCHAIN	joining chain of multimeric IgA and IgM [Source:HGNC Symbol;Acc:HGNC:5713]
ENSG00000118257	6,3	3,90E-05	NRP2	neuropilin 2 [Source:HGNC Symbol;Acc:HGNC:8005]
ENSG00000088882	6,2	7,92E-05	CPXM1	carboxypeptidase X, M14 family member 1 [Source:HGNC Symbol;Acc:HGNC:15771]
ENSG00000248176	6,2	6,96E-04		
ENSG00000156804	6,1	1,87E-07	FBXO32	F-box protein 32 [Source:HGNC Symbol;Acc:HGNC:16731]
ENSG00000244242	6,1	2,17E-07	IFITM10	interferon induced transmembrane protein 10 [Source:HGNC Symbol;Acc:HGNC:40022]
ENSG00000145685	6,1	2,93E-06	LHFPL2	LHFPL tetraspan subfamily member 2 [Source:HGNC Symbol;Acc:HGNC:6588]
ENSG00000085733	6,0	3,71E-05	CTTN	cortactin [Source:HGNC Symbol;Acc:HGNC:3338]
ENSG00000109943	6,0	1,04E-05	CRTAM	cytotoxic and regulatory T cell molecule [Source:HGNC Symbol;Acc:HGNC:24313]
ENSG00000236324	6,0	2,45E-05		
ENSG00000137441	6,0	4,23E-05	FGFBP2	fibroblast growth factor binding protein 2 [Source:HGNC Symbol;Acc:HGNC:29451]
ENSG00000143869	6,0	1,04E-05	GDF7	growth differentiation factor 7 [Source:HGNC Symbol;Acc:HGNC:4222]
ENSG00000145113	5,9	5,02E-06	MUC4	mucin 4, cell surface associated [Source:HGNC Symbol;Acc:HGNC:7514]
ENSG00000160883	5,8	7,08E-05	HK3	hexokinase 3 [Source:HGNC Symbol;Acc:HGNC:4925]
ENSG00000120278	5,7	6,14E-03	PLEKHG1	pleckstrin homology and RhoGEF domain containing G1 [Acc:HGNC:20884]
ENSG00000113555	5,7	3,58E-06	PCDH12	protocadherin 12 [Source:HGNC Symbol;Acc:HGNC:8657]
ENSG00000174885	5,7	2,96E-06	NLRP6	NLR family pyrin domain containing 6 [Source:HGNC Symbol;Acc:HGNC:22944]
ENSG00000261707	5,6	4,80E-03		
ENSG00000244265	5,6	2,38E-05	SIAH2-AS1	SIAH2 antisense RNA 1 [Source:HGNC Symbol;Acc:HGNC:40526]
ENSG00000116299	5,6	2,54E-08	KIAA1324	KIAA1324 [Source:HGNC Symbol;Acc:HGNC:29618]
ENSG00000177426	5,6	2,26E-07	TGIF1	TGFB induced factor homeobox 1 [Source:HGNC Symbol;Acc:HGNC:11776]
ENSG00000185668	5,5	2,13E-06	POU3F1	POU class 3 homeobox 1 [Source:HGNC Symbol;Acc:HGNC:9214]
ENSG00000225899	5,5	2,02E-03	FRG2B	FSHD region gene 2 family member B [Source:HGNC Symbol;Acc:HGNC:33518]
ENSG00000235125	5,5	2,79E-02	NFKBIL1	NFKB inhibitor like 1 [Source:HGNC Symbol;Acc:HGNC:7800]
ENSG00000187479	5,5	5,82E-05	C11orf96	chromosome 11 open reading frame 96 [Source:HGNC Symbol;Acc:HGNC:38675]
ENSG00000230753	5,5	4,62E-04	ZNF341-AS1	ZNF341 antisense RNA 1 [Source:HGNC Symbol;Acc:HGNC:50736]
ENSG00000236528	5,5	2,32E-03		
ENSG00000272862	5,5	4,16E-05		
ENSG00000272486	5,4	7,72E-04		
ENSG00000174944	5,4	2,29E-05	P2RY14	purinergic receptor P2Y14 [Source:HGNC Symbol;Acc:HGNC:16442]
ENSG00000259251	5,4	1,47E-04		
ENSG00000102174	5,3	1,83E-05	PHEX	phosphate regulating endopeptidase homolog X-linked [Acc:HGNC:8918]
ENSG00000143185	5,3	7,57E-04	XCL2	X-C motif chemokine ligand 2 [Source:HGNC Symbol;Acc:HGNC:10646]
ENSG00000142102	5,2	1,43E-05	PGGHG	protein-glucosylgalactosylhydroxylysine glucosidase [Acc:HGNC:26210]
ENSG00000177494	5,1	5,78E-07	ZBED2	zinc finger BED-type containing 2 [Source:HGNC Symbol;Acc:HGNC:20710]
ENSG00000259948	5,1	6,08E-03		
ENSG00000188051	5,1	3,50E-05	TMEM221	transmembrane protein 221 [Source:HGNC Symbol;Acc:HGNC:21943]
ENSG00000127318	5,1	1,42E-04	IL22	interleukin 22 [Source:HGNC Symbol;Acc:HGNC:14900]
ENSG00000180834	5,0	1,33E-04	MAP6D1	MAP6 domain containing 1 [Source:HGNC Symbol;Acc:HGNC:25753]
ENSG00000231621	5,0	9,65E-06		
ENSG00000225400	5,0	2,45E-05	RAB28P5	RAB28, member RAS oncogene family pseudogene 5 [Acc:HGNC:51547]
ENSG00000117560	5,0	4,03E-07	FASLG	Fas ligand [Source:HGNC Symbol;Acc:HGNC:11936]
ENSG00000164938	5,0	6,57E-07	TP53INP1	tumor protein p53 inducible nuclear protein 1 [Source:HGNC Symbol;Acc:HGNC:18022]
ENSG00000228216	4,9	1,45E-04		
ENSG00000068781	4,9	3,56E-03	STON1-GTF2A1L	STON1-GTF2A1L readthrough [Source:HGNC Symbol;Acc:HGNC:30651]
ENSG00000244405	4,9	1,29E-04	ETV5	ETS variant 5 [Source:HGNC Symbol;Acc:HGNC:3494]
ENSG00000167244	4,9	1,50E-03	IGF2	insulin like growth factor 2 [Source:HGNC Symbol;Acc:HGNC:5466]
ENSG00000182580	4,9	9,73E-06	EPHB3	EPH receptor B3 [Source:HGNC Symbol;Acc:HGNC:3394]
ENSG00000229502	4,9	8,49E-05		
ENSG00000113520	4,8	1,10E-05	IL4	interleukin 4 [Source:HGNC Symbol;Acc:HGNC:6014]
ENSG00000049249	4,8	3,26E-05	TNFRSF9	TNF receptor superfamily member 9 [Source:HGNC Symbol;Acc:HGNC:11924]
ENSG00000198794	4,8	2,21E-05	SCAMP5	secretory carrier membrane protein 5 [Source:HGNC Symbol;Acc:HGNC:30386]
ENSG00000265787	4,7	4,21E-04	CYP4F35P	cytochrome P450 family 4 subfamily F member 35, pseudogene [Acc:HGNC:39954]
ENSG00000135862	4,7	2,57E-04	LAMC1	laminin subunit gamma 1 [Source:HGNC Symbol;Acc:HGNC:6492]
ENSG00000198574	4,7	3,86E-02	SH2D1B	SH2 domain containing 1B [Source:HGNC Symbol;Acc:HGNC:30416]
ENSG00000103196	4,7	8,25E-06	CRISPLD2	cysteine rich secretory protein LCCL domain containing 2 [Acc:HGNC:25248]
ENSG00000123358	4,6	4,98E-06	NR4A1	nuclear receptor subfamily 4 group A member 1 [Source:HGNC Symbol;Acc:HGNC:7980]
ENSG00000185634	4,6	8,12E-07	SHC4	SHC adaptor protein 4 [Source:HGNC Symbol;Acc:HGNC:16743]
ENSG00000206013	4,6	7,38E-04	IFITM5	interferon induced transmembrane protein 5 [Source:HGNC Symbol;Acc:HGNC:16644]
ENSG00000182397	4,6	2,03E-06	DNM1P46	dynamin 1 pseudogene 46 [Source:HGNC Symbol;Acc:HGNC:35199]
ENSG00000172380	4,5	2,27E-04	GNG12	G protein subunit gamma 12 [Source:HGNC Symbol;Acc:HGNC:19663]
ENSG00000204172	4,5	6,33E-03	AGAP9	ArfGAP with GTPase domain, ankyrin repeat and PH domain 9 [Acc:HGNC:23463]
ENSG00000266642	4,5	8,52E-03		
ENSG00000135318	4,5	5,82E-05	NT5E	5'-nucleotidase ecto [Source:HGNC Symbol;Acc:HGNC:8021]
ENSG00000165152	4,5	2,08E-04	TMEM246	transmembrane protein 246 [Source:HGNC Symbol;Acc:HGNC:28180]
ENSG00000234261	4,5	1,08E-05		
ENSG00000132170	4,5	1,39E-06	PPARG	peroxisome proliferator activated receptor gamma [Source:HGNC Symbol;Acc:HGNC:9236]
ENSG00000234361	4,5	3,35E-04		
ENSG00000168243	4,4	1,04E-05	GNG4	G protein subunit gamma 4 [Source:HGNC Symbol;Acc:HGNC:4407]
ENSG00000116991	4,4	3,16E-05	SIPA1L2	signal induced proliferation associated 1 like 2 [Source:HGNC Symbol;Acc:HGNC:23800]
ENSG00000186197	4,4	6,97E-06	EDARADD	EDAR associated death domain [Source:HGNC Symbol;Acc:HGNC:14341]
ENSG00000108511	4,4	1,81E-05	HOXB6	homeobox B6 [Source:HGNC Symbol;Acc:HGNC:5117]
ENSG00000089692	4,4	4,51E-07	LAG3	lymphocyte activating 3 [Source:HGNC Symbol;Acc:HGNC:6476]
ENSG00000119411	4,4	1,15E-05	BSPRY	B-box and SPRY domain containing [Source:HGNC Symbol;Acc:HGNC:18232]
ENSG00000249626	4,3	1,26E-05		
ENSG00000231651	4,3	5,53E-06	DLG3-AS1	DLG3 antisense RNA 1 [Source:HGNC Symbol;Acc:HGNC:40182]
ENSG00000102445	4,3	6,12E-04	RUBCNL	RUN and cysteine rich domain containing beclin 1 interacting protein like [Acc:HGNC:20420]
ENSG00000136205	4,3	3,00E-03	TNS3	tensin 3 [Source:HGNC Symbol;Acc:HGNC:21616]
ENSG00000110777	4,3	5,93E-06	POU2AF1	POU class 2 associating factor 1 [Source:HGNC Symbol;Acc:HGNC:9211]
ENSG00000148426	4,3	7,75E-05	PROSER2	proline and serine rich 2 [Source:HGNC Symbol;Acc:HGNC:23728]
ENSG00000267650	4,3	1,67E-03		
ENSG00000140859	4,2	8,92E-07	KIFC3	kinesin family member C3 [Source:HGNC Symbol;Acc:HGNC:6326]
ENSG00000122367	4,2	6,82E-03	LDB3	LIM domain binding 3 [Source:HGNC Symbol;Acc:HGNC:15710]
ENSG00000111537	4,2	2,45E-05	IFNG	interferon gamma [Source:HGNC Symbol;Acc:HGNC:5438]
ENSG00000164949	4,2	1,61E-05	GEM	GTP binding protein overexpressed in skeletal muscle [Acc:HGNC:4234]
ENSG00000258212	4,2	3,36E-03	ZNF75BP	zinc finger protein 75B, pseudogene [Source:HGNC Symbol;Acc:HGNC:13147]
ENSG00000204103	4,2	4,24E-04	MAFB	MAF bZIP transcription factor B [Source:HGNC Symbol;Acc:HGNC:6408]
ENSG00000151276	4,2	4,16E-06	MAGI1	membrane associated guanylate kinase, WW and PDZ domain containing 1 [Acc:HGNC:946]
ENSG00000153563	4,2	1,41E-03	CD8A	CD8a molecule [Source:HGNC Symbol;Acc:HGNC:1706]
ENSG00000102755	4,2	6,97E-06	FLT1	fms related tyrosine kinase 1 [Source:HGNC Symbol;Acc:HGNC:3763]
ENSG00000128253	4,2	2,51E-03	RFPL2	ret finger protein like 2 [Source:HGNC Symbol;Acc:HGNC:9979]
ENSG00000275778	4,1	2,02E-02		
ENSG00000106537	4,1	1,62E-05	TSPAN13	tetraspanin 13 [Source:HGNC Symbol;Acc:HGNC:21643]
ENSG00000198125	4,1	1,13E-03	MB	myoglobin [Source:HGNC Symbol;Acc:HGNC:6915]
ENSG00000224769	4,1	2,29E-05	MUC20P1	mucin 20, cell surface associated pseudogene 1 [Source:HGNC Symbol;Acc:HGNC:51921]
ENSG00000226260	4,1	4,07E-02	HLA-DRA	major histocompatibility complex, class II, DR alpha [Source:HGNC Symbol;Acc:HGNC:4947]
ENSG00000104081	4,1	3,19E-04	BMF	Bcl2 modifying factor [Source:HGNC Symbol;Acc:HGNC:24132]
ENSG00000050820	4,1	1,77E-05	BCAR1	BCAR1, Cas family scaffolding protein [Source:HGNC Symbol;Acc:HGNC:971]
ENSG00000230968	4,1	4,21E-04		
ENSG00000104921	4,1	1,53E-03	FCER2	Fc fragment of IgE receptor II [Source:HGNC Symbol;Acc:HGNC:3612]
ENSG00000169758	4,1	1,35E-04	TMEM266	transmembrane protein 266 [Source:HGNC Symbol;Acc:HGNC:26763]
ENSG00000213626	4,1	3,44E-07	LBH	limb bud and heart development [Source:HGNC Symbol;Acc:HGNC:29532]
ENSG00000230623	4,0	7,97E-05		
ENSG00000224360	4,0	3,76E-02	DDR1-AS1	DDR1 antisense RNA 1 (head to head) [Source:HGNC Symbol;Acc:HGNC:28694]
ENSG00000121900	4,0	1,04E-05	TMEM54	transmembrane protein 54 [Source:HGNC Symbol;Acc:HGNC:24143]
ENSG00000176641	4,0	4,24E-04	RNF152	ring finger protein 152 [Source:HGNC Symbol;Acc:HGNC:26811]
ENSG00000155926	4,0	9,51E-08	SLA	Src like adaptor [Source:HGNC Symbol;Acc:HGNC:10902]
ENSG00000107242	4,0	1,18E-05	PIP5K1B	phosphatidylinositol-4-phosphate 5-kinase type 1 beta [Acc:HGNC:8995]
ENSG00000124191	4,0	4,07E-05	TOX2	TOX high mobility group box family member 2 [Source:HGNC Symbol;Acc:HGNC:16095]
ENSG00000147041	4,0	8,15E-04	SYTL5	synaptotagmin like 5 [Source:HGNC Symbol;Acc:HGNC:15589]
ENSG00000273275	4,0	1,32E-03		
ENSG00000091972	4,0	3,33E-04	CD200	CD200 molecule [Source:HGNC Symbol;Acc:HGNC:7203]
ENSG00000258760	4,0	2,01E-05		
ENSG00000020577	4,0	3,39E-05	SAMD4A	sterile alpha motif domain containing 4A [Source:HGNC Symbol;Acc:HGNC:23023]
ENSG00000164023	3,9	7,95E-06	SGMS2	sphingomyelin synthase 2 [Source:HGNC Symbol;Acc:HGNC:28395]
ENSG00000215045	3,9	4,31E-06	GRID2IP	Grid2 interacting protein [Source:HGNC Symbol;Acc:HGNC:18464]
ENSG00000223687	3,9	2,81E-02	ZNF311	zinc finger protein 311 [Source:HGNC Symbol;Acc:HGNC:13847]
ENSG00000253396	3,9	4,49E-05		
ENSG00000283294	3,9	5,06E-03		
ENSG00000109321	3,9	3,22E-04	AREG	amphiregulin [Source:HGNC Symbol;Acc:HGNC:651]
ENSG00000269889	3,9	2,49E-03		
ENSG00000225194	3,9	2,71E-06	LINC00092	long intergenic non-protein coding RNA 92 [Source:HGNC Symbol;Acc:HGNC:31408]
ENSG00000273320	3,9	1,96E-04		
ENSG00000101384	3,9	5,44E-06	JAG1	jagged 1 [Source:HGNC Symbol;Acc:HGNC:6188]
ENSG00000140876	3,9	2,68E-05	NUDT7	nudix hydrolase 7 [Source:HGNC Symbol;Acc:HGNC:8054]
ENSG00000210194	3,9	2,16E-04	MT-TE	mitochondrially encoded tRNA glutamic acid [Source:HGNC Symbol;Acc:HGNC:7479]
ENSG00000246130	3,9	1,33E-04		
ENSG00000157150	3,8	2,03E-03	TIMP4	TIMP metallopeptidase inhibitor 4 [Source:HGNC Symbol;Acc:HGNC:11823]
ENSG00000259003	3,8	1,54E-04		
ENSG00000160318	3,8	4,98E-06	CLDND2	claudin domain containing 2 [Source:HGNC Symbol;Acc:HGNC:28511]
ENSG00000087842	3,8	1,41E-07	PIR	pirin [Source:HGNC Symbol;Acc:HGNC:30048]
ENSG00000198576	3,8	8,91E-04	ARC	activity regulated cytoskeleton associated protein [Source:HGNC Symbol;Acc:HGNC:648]
ENSG00000274869	3,8	2,00E-04	-	-
ENSG00000109684	3,8	3,13E-05	CLNK	cytokine dependent hematopoietic cell linker [Source:HGNC Symbol;Acc:HGNC:17438]
ENSG00000266076	3,8	9,30E-03		
ENSG00000171408	3,8	1,58E-05	PDE7B	phosphodiesterase 7B [Source:HGNC Symbol;Acc:HGNC:8792]
ENSG00000175170	3,8	2,74E-05	FAM182B	family with sequence similarity 182 member B [Source:HGNC Symbol;Acc:HGNC:34503]
ENSG00000268257	3,8	7,71E-04	AIRN	antisense of IGF2R non-protein coding RNA [Source:HGNC Symbol;Acc:HGNC:34515]
ENSG00000271662	3,8	4,02E-03		
ENSG00000228919	3,8	1,45E-03		
ENSG00000173762	3,7	6,82E-06	CD7	CD7 molecule [Source:HGNC Symbol;Acc:HGNC:1695]
ENSG00000081377	3,7	3,65E-06	CDC14B	cell division cycle 14B [Source:HGNC Symbol;Acc:HGNC:1719]
ENSG00000073150	3,7	1,10E-06	PANX2	pannexin 2 [Source:HGNC Symbol;Acc:HGNC:8600]
ENSG00000235141	3,7	1,35E-03	COX6CP17	cytochrome c oxidase subunit 6C pseudogene 17 [Source:HGNC Symbol;Acc:HGNC:49369]
ENSG00000205710	3,7	1,10E-06	C17orf107	chromosome 17 open reading frame 107 [Source:HGNC Symbol;Acc:HGNC:37238]
ENSG00000268355	3,7	7,42E-05		
ENSG00000266145	3,7	3,55E-04	RHOT1P1	ras homolog family member T1 pseudogene 1 [Source:HGNC Symbol;Acc:HGNC:23777]
ENSG00000236494	3,7	2,73E-04		
ENSG00000225079	3,7	1,68E-06	FTH1P22	ferritin heavy chain 1 pseudogene 22 [Source:HGNC Symbol;Acc:HGNC:37640]
ENSG00000125657	3,7	3,64E-07	TNFSF9	TNF superfamily member 9 [Source:HGNC Symbol;Acc:HGNC:11939]
ENSG00000283187	3,7	4,02E-03	-	-
ENSG00000243968	3,6	4,45E-03	RN7SL402P	RNA, 7SL, cytoplasmic 402, pseudogene [Source:HGNC Symbol;Acc:HGNC:46418]
ENSG00000054598	3,6	9,64E-05	FOXC1	forkhead box C1 [Source:HGNC Symbol;Acc:HGNC:3800]
ENSG00000103855	3,6	4,39E-05	CD276	CD276 molecule [Source:HGNC Symbol;Acc:HGNC:19137]
ENSG00000129757	3,6	3,33E-05	CDKN1C	cyclin dependent kinase inhibitor 1C [Source:HGNC Symbol;Acc:HGNC:1786]
ENSG00000130340	3,6	5,01E-07	SNX9	sorting nexin 9 [Source:HGNC Symbol;Acc:HGNC:14973]
ENSG00000261449	3,6	1,45E-03		
ENSG00000228294	3,6	4,02E-02	BMS1P17	BMS1, ribosome biogenesis factor pseudogene 17 [Source:HGNC Symbol;Acc:HGNC:49162]
ENSG00000111186	3,6	1,16E-03	WNT5B	Wnt family member 5B [Source:HGNC Symbol;Acc:HGNC:16265]
ENSG00000165633	3,6	6,22E-07	VSTM4	V-set and transmembrane domain containing 4 [Source:HGNC Symbol;Acc:HGNC:26470]
ENSG00000257924	3,6	5,99E-05	LINC02416	long intergenic non-protein coding RNA 2416 [Source:HGNC Symbol;Acc:HGNC:53345]
ENSG00000113594	3,5	2,97E-04	LIFR	LIF receptor alpha [Source:HGNC Symbol;Acc:HGNC:6597]
ENSG00000105963	3,5	4,82E-07	ADAP1	ArfGAP with dual PH domains 1 [Source:HGNC Symbol;Acc:HGNC:16486]
ENSG00000114423	3,5	1,40E-06	CBLB	Cbl proto-oncogene B [Source:HGNC Symbol;Acc:HGNC:1542]
ENSG00000259658	3,5	7,10E-06		
ENSG00000268038	3,5	2,70E-04	LINC01785	long intergenic non-protein coding RNA 1785 [Source:HGNC Symbol;Acc:HGNC:25060]
ENSG00000125968	3,5	1,51E-04	ID1	inhibitor of DNA binding 1, HLH protein [Source:HGNC Symbol;Acc:HGNC:5360]
ENSG00000224363	3,5	3,48E-02		
ENSG00000246792	3,5	6,87E-04		
ENSG00000074590	3,5	9,02E-03	NUAK1	NUAK family kinase 1 [Source:HGNC Symbol;Acc:HGNC:14311]
ENSG00000145103	3,5	1,74E-04	ILDR1	immunoglobulin like domain containing receptor 1 [Source:HGNC Symbol;Acc:HGNC:28741]
ENSG00000237232	3,5	2,23E-04	ZNF295-AS1	ZNF295 antisense RNA 1 [Source:HGNC Symbol;Acc:HGNC:23130]
ENSG00000270300	3,5	4,95E-04	PHACTR2P1	phosphatase and actin regulator 2 pseudogene 1 [Source:HGNC Symbol;Acc:HGNC:49488]
ENSG00000273308	3,5	1,39E-04		
ENSG00000222032	3,5	1,48E-03		
ENSG00000139193	3,5	3,92E-06	CD27	CD27 molecule [Source:HGNC Symbol;Acc:HGNC:11922]
ENSG00000232656	3,5	4,54E-05	IDI2-AS1	IDI2 antisense RNA 1 [Source:HGNC Symbol;Acc:HGNC:30885]
ENSG00000157388	3,4	3,76E-06	CACNA1D	calcium voltage-gated channel subunit alpha1 D [Source:HGNC Symbol;Acc:HGNC:1391]
ENSG00000262136	3,4	1,72E-03		
ENSG00000120659	3,4	3,52E-04	TNFSF11	TNF superfamily member 11 [Source:HGNC Symbol;Acc:HGNC:11926]
ENSG00000183691	3,4	2,39E-04	NOG	noggin [Source:HGNC Symbol;Acc:HGNC:7866]
ENSG00000148488	3,4	1,26E-05	ST8SIA6	ST8 alpha-N-acetyl-neuraminide alpha-2,8-sialyltransferase 6 [Acc:HGNC:23317]
ENSG00000104722	3,4	1,08E-03	NEFM	neurofilament medium [Source:HGNC Symbol;Acc:HGNC:7734]
ENSG00000167037	3,4	8,02E-06	SGSM1	small G protein signaling modulator 1 [Source:HGNC Symbol;Acc:HGNC:29410]
ENSG00000128040	3,4	3,36E-03	SPINK2	serine peptidase inhibitor, Kazal type 2 [Source:HGNC Symbol;Acc:HGNC:11245]
ENSG00000232237	3,4	7,36E-04	ASCL5	achaete-scute family bHLH transcription factor 5 [Source:HGNC Symbol;Acc:HGNC:33169]
ENSG00000235674	3,4	2,47E-03	LDHAP2	lactate dehydrogenase A pseudogene 2 [Source:HGNC Symbol;Acc:HGNC:6537]
ENSG00000212329	3,4	1,36E-03	RNU6-316P	RNA, U6 small nuclear 316, pseudogene [Source:HGNC Symbol;Acc:HGNC:47279]
ENSG00000124006	3,4	3,96E-03	OBSL1	obscurin like 1 [Source:HGNC Symbol;Acc:HGNC:29092]
ENSG00000253557	3,4	4,20E-05		
ENSG00000272735	3,4	1,89E-04		
ENSG00000197057	3,4	6,69E-05	DTHD1	death domain containing 1 [Source:HGNC Symbol;Acc:HGNC:37261]
ENSG00000050730	3,4	6,67E-05	TNIP3	TNFAIP3 interacting protein 3 [Source:HGNC Symbol;Acc:HGNC:19315]
ENSG00000158163	3,4	2,21E-03	DZIP1L	DAZ interacting zinc finger protein 1 like [Source:HGNC Symbol;Acc:HGNC:26551]
ENSG00000158186	3,3	1,73E-04	MRAS	muscle RAS oncogene homolog [Source:HGNC Symbol;Acc:HGNC:7227]
ENSG00000254835	3,3	2,31E-02	RNF185-AS1	RNF185 antisense RNA 1 [Source:HGNC Symbol;Acc:HGNC:41161]
ENSG00000146267	3,3	5,77E-05	FAXC	failed axon connections homolog [Source:HGNC Symbol;Acc:HGNC:20742]
ENSG00000264695	3,3	2,52E-02		
ENSG00000222022	3,3	7,64E-03		
ENSG00000232162	3,3	7,99E-04	USP12-AS1	USP12 antisense RNA 1 [Source:HGNC Symbol;Acc:HGNC:39961]
ENSG00000220867	3,3	2,84E-03	HSPE1P26	heat shock protein family E (Hsp10) member 1 pseudogene 26 [Acc:HGNC:49345]
ENSG00000100156	3,3	5,91E-03	SLC16A8	solute carrier family 16 member 8 [Source:HGNC Symbol;Acc:HGNC:16270]
ENSG00000144481	3,3	8,04E-05	TRPM8	transient receptor potential cation channel subfamily M member 8 [Acc:HGNC:17961]
ENSG00000204754	3,3	6,10E-04	LINC01951	long intergenic non-protein coding RNA 1951 [Source:HGNC Symbol;Acc:HGNC:52774]
ENSG00000114638	3,3	2,26E-04	UPK1B	uroplakin 1B [Source:HGNC Symbol;Acc:HGNC:12578]
ENSG00000253878	3,3	1,46E-04		
ENSG00000245848	3,3	7,73E-06	CEBPA	CCAAT/enhancer binding protein alpha [Source:HGNC Symbol;Acc:HGNC:1833]
ENSG00000265118	3,3	1,84E-03		
ENSG00000104450	3,3	2,54E-08	SPAG1	sperm associated antigen 1 [Source:HGNC Symbol;Acc:HGNC:11212]
ENSG00000188672	3,3	1,37E-04	RHCE	Rh blood group CcEe antigens [Source:HGNC Symbol;Acc:HGNC:10008]
ENSG00000213171	3,3	2,66E-05	LINGO4	leucine rich repeat and Ig domain containing 4 [Source:HGNC Symbol;Acc:HGNC:31814]
ENSG00000230965	3,3	9,05E-03	SNX18P13	sorting nexin 18 pseudogene 13 [Source:HGNC Symbol;Acc:HGNC:39621]
ENSG00000144893	3,3	1,50E-05	MED12L	mediator complex subunit 12 like [Source:HGNC Symbol;Acc:HGNC:16050]
ENSG00000227231	3,3	1,95E-04	IER3	immediate early response 3 [Source:HGNC Symbol;Acc:HGNC:5392]
ENSG00000230128	3,3	1,95E-04	IER3	immediate early response 3 [Source:HGNC Symbol;Acc:HGNC:5392]
ENSG00000235030	3,3	1,95E-04	IER3	immediate early response 3 [Source:HGNC Symbol;Acc:HGNC:5392]
ENSG00000237155	3,3	1,95E-04	IER3	immediate early response 3 [Source:HGNC Symbol;Acc:HGNC:5392]
ENSG00000115009	3,3	2,84E-05	CCL20	C-C motif chemokine ligand 20 [Source:HGNC Symbol;Acc:HGNC:10619]
ENSG00000225511	3,3	2,51E-04	LINC00475	long intergenic non-protein coding RNA 475 [Source:HGNC Symbol;Acc:HGNC:23569]
ENSG00000260101	3,3	8,00E-05		
ENSG00000238390	3,3	1,54E-03		
ENSG00000261186	3,3	1,71E-03	LINC01238	long intergenic non-protein coding RNA 1238 [Source:HGNC Symbol;Acc:HGNC:49795]
ENSG00000101230	3,2	6,78E-04	ISM1	isthmin 1 [Source:HGNC Symbol;Acc:HGNC:16213]
ENSG00000185442	3,2	7,69E-07	FAM174B	family with sequence similarity 174 member B [Source:HGNC Symbol;Acc:HGNC:34339]
ENSG00000188163	3,2	1,62E-05	FAM166A	family with sequence similarity 166 member A [Source:HGNC Symbol;Acc:HGNC:33818]
ENSG00000196218	3,2	2,24E-04	RYR1	ryanodine receptor 1 [Source:HGNC Symbol;Acc:HGNC:10483]
ENSG00000112238	3,2	2,32E-04	PRDM13	PR/SET domain 13 [Source:HGNC Symbol;Acc:HGNC:13998]
ENSG00000162576	3,2	5,19E-07	MXRA8	matrix remodeling associated 8 [Source:HGNC Symbol;Acc:HGNC:7542]
ENSG00000170412	3,2	1,08E-04	GPRC5C	G protein-coupled receptor class C group 5 member C [Acc:HGNC:13309]
ENSG00000121743	3,2	7,58E-04	GJA3	gap junction protein alpha 3 [Source:HGNC Symbol;Acc:HGNC:4277]
ENSG00000095637	3,2	4,06E-06	SORBS1	sorbin and SH3 domain containing 1 [Source:HGNC Symbol;Acc:HGNC:14565]
ENSG00000227145	3,2	1,68E-04	IL21-AS1	IL21 antisense RNA 1 [Source:HGNC Symbol;Acc:HGNC:40299]
ENSG00000049130	3,2	7,40E-03	KITLG	KIT ligand [Source:HGNC Symbol;Acc:HGNC:6343]
ENSG00000254693	3,2	2,03E-03		
ENSG00000229178	3,2	4,88E-05		
ENSG00000163359	3,2	2,50E-03	COL6A3	collagen type VI alpha 3 chain [Source:HGNC Symbol;Acc:HGNC:2213]
ENSG00000179841	3,2	2,91E-06	AKAP5	A-kinase anchoring protein 5 [Source:HGNC Symbol;Acc:HGNC:375]
ENSG00000166145	3,2	1,34E-05	SPINT1	serine peptidase inhibitor, Kunitz type 1 [Source:HGNC Symbol;Acc:HGNC:11246]
ENSG00000050165	3,2	8,02E-03	DKK3	dickkopf WNT signaling pathway inhibitor 3 [Source:HGNC Symbol;Acc:HGNC:2893]
ENSG00000164972	3,2	1,60E-03	C9orf24	chromosome 9 open reading frame 24 [Source:HGNC Symbol;Acc:HGNC:19919]
ENSG00000143669	3,2	7,52E-06	LYST	lysosomal trafficking regulator [Source:HGNC Symbol;Acc:HGNC:1968]
ENSG00000185650	3,2	2,23E-05	ZFP36L1	ZFP36 ring finger protein like 1 [Source:HGNC Symbol;Acc:HGNC:1107]
ENSG00000124216	3,2	7,23E-04	SNAI1	snail family transcriptional repressor 1 [Source:HGNC Symbol;Acc:HGNC:11128]
ENSG00000255819	3,2	3,08E-02	KLRC4-KLRK1	KLRC4-KLRK1 readthrough [Source:HGNC Symbol;Acc:HGNC:48357]
ENSG00000267690	3,2	2,58E-02	LDLRAD4-AS1	LDLRAD4 antisense RNA 1 [Source:HGNC Symbol;Acc:HGNC:48592]
ENSG00000223947	3,2	4,99E-03		
ENSG00000177034	3,1	2,54E-08	MTX3	metaxin 3 [Source:HGNC Symbol;Acc:HGNC:24812]
ENSG00000230148	3,1	3,30E-04	HOXB-AS1	HOXB cluster antisense RNA 1 [Source:HGNC Symbol;Acc:HGNC:43744]
ENSG00000167549	3,1	1,37E-04	CORO6	coronin 6 [Source:HGNC Symbol;Acc:HGNC:21356]
ENSG00000179403	3,1	1,11E-02	VWA1	von Willebrand factor A domain containing 1 [Source:HGNC Symbol;Acc:HGNC:30910]
ENSG00000235641	3,1	1,42E-05	LINC00484	long intergenic non-protein coding RNA 484 [Source:HGNC Symbol;Acc:HGNC:27862]
ENSG00000109832	3,1	1,34E-03	DDX25	DEAD-box helicase 25 [Source:HGNC Symbol;Acc:HGNC:18698]
ENSG00000260196	3,1	1,24E-02		
ENSG00000245552	3,1	5,14E-04		
ENSG00000260782	3,1	4,23E-03		
ENSG00000133069	3,1	6,22E-07	TMCC2	transmembrane and coiled-coil domain family 2 [Source:HGNC Symbol;Acc:HGNC:24239]
ENSG00000151468	3,1	1,31E-03	CCDC3	coiled-coil domain containing 3 [Source:HGNC Symbol;Acc:HGNC:23813]
ENSG00000240254	3,1	3,77E-04	B4GALT4-AS1	B4GALT4 antisense RNA 1 [Source:HGNC Symbol;Acc:HGNC:40090]
ENSG00000260805	3,1	4,25E-04		
ENSG00000137101	3,1	5,98E-06	CD72	CD72 molecule [Source:HGNC Symbol;Acc:HGNC:1696]
ENSG00000253842	3,1	4,16E-03		
ENSG00000061676	3,1	9,73E-07	NCKAP1	NCK associated protein 1 [Source:HGNC Symbol;Acc:HGNC:7666]
ENSG00000188211	3,1	3,75E-04	NCR3LG1	natural killer cell cytotoxicity receptor 3 ligand 1 [Source:HGNC Symbol;Acc:HGNC:42400]
ENSG00000231435	3,1	1,21E-03		
ENSG00000257947	3,1	1,09E-04		
ENSG00000166046	3,1	6,37E-06	TCP11L2	t-complex 11 like 2 [Source:HGNC Symbol;Acc:HGNC:28627]
ENSG00000272631	3,1	4,84E-04		
ENSG00000228570	3,1	1,33E-02	NUTM2E	NUT family member 2E [Source:HGNC Symbol;Acc:HGNC:23448]
ENSG00000241713	3,1	3,44E-02	LY6G5B	lymphocyte antigen 6 family member G5B [Source:HGNC Symbol;Acc:HGNC:13931]
ENSG00000188389	3,1	3,64E-07	PDCD1	programmed cell death 1 [Source:HGNC Symbol;Acc:HGNC:8760]
ENSG00000267334	3,1	2,83E-04		
ENSG00000231062	3,1	9,76E-04		
ENSG00000134531	3,1	2,54E-08	EMP1	epithelial membrane protein 1 [Source:HGNC Symbol;Acc:HGNC:3333]
ENSG00000182168	3,1	1,53E-02	UNC5C	unc-5 netrin receptor C [Source:HGNC Symbol;Acc:HGNC:12569]
ENSG00000236304	3,1	1,92E-02		
ENSG00000167604	3,1	2,81E-05	NFKBID	NFKB inhibitor delta [Source:HGNC Symbol;Acc:HGNC:15671]
ENSG00000253666	3,1	1,43E-03		
ENSG00000272398	3,1	6,84E-04	CD24	CD24 molecule [Source:HGNC Symbol;Acc:HGNC:1645]
ENSG00000178093	3,1	4,11E-02	TSSK6	testis specific serine kinase 6 [Source:HGNC Symbol;Acc:HGNC:30410]
ENSG00000170485	3,1	3,01E-04	NPAS2	neuronal PAS domain protein 2 [Source:HGNC Symbol;Acc:HGNC:7895]
ENSG00000196422	3,1	3,59E-06	PPP1R26	protein phosphatase 1 regulatory subunit 26 [Source:HGNC Symbol;Acc:HGNC:29089]
ENSG00000122862	3,1	2,01E-06	SRGN	serglycin [Source:HGNC Symbol;Acc:HGNC:9361]
ENSG00000149212	3,1	5,40E-04	SESN3	sestrin 3 [Source:HGNC Symbol;Acc:HGNC:23060]
ENSG00000122335	3,1	1,73E-05	SERAC1	serine active site containing 1 [Source:HGNC Symbol;Acc:HGNC:21061]
ENSG00000007237	3,1	1,36E-02	GAS7	growth arrest specific 7 [Source:HGNC Symbol;Acc:HGNC:4169]
ENSG00000234377	3,0	3,61E-03	RNF219-AS1	RNF219 antisense RNA 1 [Source:HGNC Symbol;Acc:HGNC:42700]
ENSG00000270846	3,0	6,35E-03		
ENSG00000253320	3,0	2,30E-06	AZIN1-AS1	AZIN1 antisense RNA 1 [Source:HGNC Symbol;Acc:HGNC:51558]
ENSG00000227375	3,0	1,15E-04	DLG1-AS1	DLG1 antisense RNA 1 [Source:HGNC Symbol;Acc:HGNC:44154]
ENSG00000211855	3,0	5,20E-05	TRAJ34	T cell receptor alpha joining 34 [Source:HGNC Symbol;Acc:HGNC:12064]
ENSG00000213073	3,0	2,45E-05		
ENSG00000232530	3,0	2,07E-03	LIF-AS1	LIF antisense RNA 1 [Source:HGNC Symbol;Acc:HGNC:53473]
ENSG00000140948	3,0	6,70E-06	ZCCHC14	zinc finger CCHC-type containing 14 [Source:HGNC Symbol;Acc:HGNC:24134]
ENSG00000105672	3,0	5,75E-06	ETV2	ETS variant 2 [Source:HGNC Symbol;Acc:HGNC:3491]
ENSG00000164692	3,0	8,79E-03	COL1A2	collagen type I alpha 2 chain [Source:HGNC Symbol;Acc:HGNC:2198]
ENSG00000258922	3,0	1,95E-03		
ENSG00000166689	3,0	1,43E-05	PLEKHA7	pleckstrin homology domain containing A7 [Source:HGNC Symbol;Acc:HGNC:27049]
ENSG00000262481	3,0	4,92E-03	TMEM256-PLSC	TMEM256-PLSCR3 readthrough (NMD candidate) [Source:HGNC Symbol;Acc:HGNC:49186]
ENSG00000237400	3,0	5,50E-03		
ENSG00000101405	3,0	5,73E-03	OXT	oxytocin/neurophysin I prepropeptide [Source:HGNC Symbol;Acc:HGNC:8528]
ENSG00000154269	3,0	3,95E-04	ENPP3	ectonucleotide pyrophosphatase/phosphodiesterase 3 [Acc:HGNC:3358]
ENSG00000138271	3,0	3,14E-03	GPR87	G protein-coupled receptor 87 [Source:HGNC Symbol;Acc:HGNC:4538]
ENSG00000196358	3,0	1,20E-03	NTNG2	netrin G2 [Source:HGNC Symbol;Acc:HGNC:14288]
ENSG00000258465	3,0	1,82E-02		
ENSG00000251682	3,0	1,60E-03		

**Supplementary Table 2:** Transcripts down-regulated by T0070907 in memory CCR6+ T-cells

Gene ID	FC	Adj. P	Gene symbol	Gene description
ENSG00000203772	-8,71	1,25E-02	SPRN	shadow of prion protein [Source:HGNC Symbol;Acc:HGNC:16871]
ENSG00000134326	-8,51	7,83E-06	CMPK2	cytidine/uridine monophosphate kinase 2 [Source:HGNC Symbol;Acc:HGNC:27015]
ENSG00000163464	-8,10	2,92E-05	CXCR1	C-X-C motif chemokine receptor 1 [Source:HGNC Symbol;Acc:HGNC:6026]
ENSG00000134321	-8,09	2,20E-05	RSAD2	radical S-adenosyl methionine domain containing 2 [Source:HGNC Symbol;Acc:HGNC:30908]
ENSG00000133101	-8,07	1,35E-03	CCNA1	cyclin A1 [Source:HGNC Symbol;Acc:HGNC:1577]
ENSG00000145649	-7,39	9,98E-06	GZMA	granzyme A [Source:HGNC Symbol;Acc:HGNC:4708]
ENSG00000251349	-7,15	2,55E-02	MSANTD3-TMEFF1	MSANTD3-TMEFF1 readthrough [Source:HGNC Symbol;Acc:HGNC:38838]
ENSG00000262655	-6,52	5,63E-05	SPON1	spondin 1 [Source:HGNC Symbol;Acc:HGNC:11252]
ENSG00000144476	-6,50	2,01E-06	ACKR3	atypical chemokine receptor 3 [Source:HGNC Symbol;Acc:HGNC:23692]
ENSG00000132359	-6,02	3,59E-06	RAP1GAP2	RAP1 GTPase activating protein 2 [Source:HGNC Symbol;Acc:HGNC:29176]
ENSG00000137959	-5,98	7,24E-05	IFI44L	interferon induced protein 44 like [Source:HGNC Symbol;Acc:HGNC:17817]
ENSG00000188822	-5,40	1,22E-02	CNR2	cannabinoid receptor 2 [Source:HGNC Symbol;Acc:HGNC:2160]
ENSG00000273217	-5,14	1,39E-02		
ENSG00000227422	-4,96	1,28E-02	DDR1-AS1	DDR1 antisense RNA 1 (head to head) [Source:HGNC Symbol;Acc:HGNC:28694]
ENSG00000184979	-4,94	2,34E-05	USP18	ubiquitin specific peptidase 18 [Source:HGNC Symbol;Acc:HGNC:12616]
ENSG00000116574	-4,84	7,73E-06	RHOU	ras homolog family member U [Source:HGNC Symbol;Acc:HGNC:17794]
ENSG00000133321	-4,81	3,60E-07	RARRES3	retinoic acid receptor responder 3 [Source:HGNC Symbol;Acc:HGNC:9869]
ENSG00000203896	-4,80	2,61E-05	LIME1	Lck interacting transmembrane adaptor 1 [Source:HGNC Symbol;Acc:HGNC:26016]
ENSG00000164342	-4,76	8,32E-06	TLR3	toll like receptor 3 [Source:HGNC Symbol;Acc:HGNC:11849]
ENSG00000185745	-4,74	2,45E-05	IFIT1	interferon induced protein with tetratricopeptide repeats 1 [Source:HGNC Symbol;Acc:HGNC:5407]
ENSG00000079385	-4,63	8,16E-07	CEACAM1	carcinoembryonic antigen related cell adhesion molecule 1 [Source:HGNC Symbol;Acc:HGNC:1814]
ENSG00000114455	-4,49	9,13E-06	HHLA2	HERV-H LTR-associating 2 [Source:HGNC Symbol;Acc:HGNC:4905]
ENSG00000182585	-4,47	2,54E-04	EPGN	epithelial mitogen [Source:HGNC Symbol;Acc:HGNC:17470]
ENSG00000122043	-4,46	7,09E-05	LINC00544	long intergenic non-protein coding RNA 544 [Source:HGNC Symbol;Acc:HGNC:43679]
ENSG00000138722	-4,44	1,54E-05	MMRN1	multimerin 1 [Source:HGNC Symbol;Acc:HGNC:7178]
ENSG00000157601	-4,28	9,31E-06	MX1	MX dynamin like GTPase 1 [Source:HGNC Symbol;Acc:HGNC:7532]
ENSG00000271288	-4,19	9,89E-05	IGHV1OR15-3	immunoglobulin heavy variable 1/OR15-3 (pseudogene) [Source:HGNC Symbol;Acc:HGNC:5565]
ENSG00000132832	-4,13	3,36E-04		
ENSG00000119917	-4,01	3,02E-05	IFIT3	interferon induced protein with tetratricopeptide repeats 3 [Source:HGNC Symbol;Acc:HGNC:5411]
ENSG00000141161	-4,00	1,88E-02	UNC45B	unc-45 myosin chaperone B [Source:HGNC Symbol;Acc:HGNC:14304]
ENSG00000005102	-3,94	1,56E-04	MEOX1	mesenchyme homeobox 1 [Source:HGNC Symbol;Acc:HGNC:7013]
ENSG00000198133	-3,91	5,42E-06	TMEM229B	transmembrane protein 229B [Source:HGNC Symbol;Acc:HGNC:20130]
ENSG00000248871	-3,88	4,23E-02	TNFSF12-TNFSF13	TNFSF12-TNFSF13 readthrough [Source:HGNC Symbol;Acc:HGNC:33537]
ENSG00000278139	-3,83	1,70E-02		
ENSG00000171595	-3,73	7,95E-05	DNAI2	dynein axonemal intermediate chain 2 [Source:HGNC Symbol;Acc:HGNC:18744]
ENSG00000228913	-3,69	4,63E-02	UBD	ubiquitin D [Source:HGNC Symbol;Acc:HGNC:18795]
ENSG00000119922	-3,67	1,37E-06	IFIT2	interferon induced protein with tetratricopeptide repeats 2 [Source:HGNC Symbol;Acc:HGNC:5409]
ENSG00000183662	-3,67	1,12E-03	FAM19A1	family with sequence similarity 19 member A1, C-C motif chemokine like [Acc:HGNC:21587]
ENSG00000089127	-3,62	2,24E-06	OAS1	2'-5'-oligoadenylate synthetase 1 [Source:HGNC Symbol;Acc:HGNC:8086]
ENSG00000184451	-3,61	8,70E-05	CCR10	C-C motif chemokine receptor 10 [Source:HGNC Symbol;Acc:HGNC:4474]
ENSG00000145491	-3,52	4,70E-04	ROPN1L	rhophilin associated tail protein 1 like [Source:HGNC Symbol;Acc:HGNC:24060]
ENSG00000113088	-3,47	1,65E-04	GZMK	granzyme K [Source:HGNC Symbol;Acc:HGNC:4711]
ENSG00000168961	-3,45	1,14E-05	LGALS9	galectin 9 [Source:HGNC Symbol;Acc:HGNC:6570]
ENSG00000223405	-3,27	2,24E-02	DDR1-AS1	DDR1 antisense RNA 1 (head to head) [Source:HGNC Symbol;Acc:HGNC:28694]
ENSG00000186399	-3,27	2,43E-02	GOLGA8R	golgin A8 family member R [Source:HGNC Symbol;Acc:HGNC:44407]
ENSG00000105088	-3,26	1,15E-05	OLFM2	olfactomedin 2 [Source:HGNC Symbol;Acc:HGNC:17189]
ENSG00000121807	-3,23	4,19E-06	CCR2	C-C motif chemokine receptor 2 [Source:HGNC Symbol;Acc:HGNC:1603]
ENSG00000151490	-3,22	1,17E-04	PTPRO	protein tyrosine phosphatase, receptor type O [Source:HGNC Symbol;Acc:HGNC:9678]
ENSG00000114315	-3,22	5,50E-03	HES1	hes family bHLH transcription factor 1 [Source:HGNC Symbol;Acc:HGNC:5192]
ENSG00000137965	-3,21	1,42E-05	IFI44	interferon induced protein 44 [Source:HGNC Symbol;Acc:HGNC:16938]
ENSG00000141540	-3,20	5,09E-05	TTYH2	tweety family member 2 [Source:HGNC Symbol;Acc:HGNC:13877]
ENSG00000138792	-3,19	9,41E-06	ENPEP	glutamyl aminopeptidase [Source:HGNC Symbol;Acc:HGNC:3355]
ENSG00000136514	-3,11	2,55E-06	RTP4	receptor transporter protein 4 [Source:HGNC Symbol;Acc:HGNC:23992]
ENSG00000168461	-3,10	1,15E-04	RAB31	RAB31, member RAS oncogene family [Source:HGNC Symbol;Acc:HGNC:9771]
ENSG00000107438	-3,09	2,36E-04	PDLIM1	PDZ and LIM domain 1 [Source:HGNC Symbol;Acc:HGNC:2067]
ENSG00000255221	-3,08	1,53E-05	CARD17	caspase recruitment domain family member 17 [Source:HGNC Symbol;Acc:HGNC:33827]
ENSG00000163823	-3,05	1,15E-05	CCR1	C-C motif chemokine receptor 1 [Source:HGNC Symbol;Acc:HGNC:1602]
ENSG00000187808	-3,05	6,57E-04	SOWAHD	sosondowah ankyrin repeat domain family member D [Source:HGNC Symbol;Acc:HGNC:32960]
ENSG00000231550	-3,05	7,99E-04	PTCHD3P2	patched domain containing 3 pseudogene 2 [Source:HGNC Symbol;Acc:HGNC:44946]
ENSG00000139626	-3,04	3,35E-05	ITGB7	integrin subunit beta 7 [Source:HGNC Symbol;Acc:HGNC:6162]
ENSG00000128833	-3,00	2,01E-06	MYO5C	myosin VC [Source:HGNC Symbol;Acc:HGNC:7604]

**Supplementary Table 3.** Antibodies used for flow cytometry analysis and sorting

	Fluorochrome	Clone	Vendor
CD4	AlexaFluor700	RPAT4	BD Pharmingen (San Diego, CA, USA)
CCR6	PE	11A9	
CCR5	PE	2D7/CCR5	
IFN-y	AlexaFluor700	B27	
CXCR4	PE	12G5	
Ki67	FITC	B56	
BST-2 (CD137	BV421	Y129	
B7 integrin	FITC	FIB504	eBioscience (San Diego, CA, USA)
phospho-mTOR	FITC	MRRBY	
CD56	FITC	MEM188	
IL-17A	PE	eBio64BEC17	
CD8	FITC	BW135/80	Miltenyi Biotech (Auburn, CA, USA)
CD19	FITC	LT19	
CD45RA	APCeFluor780	HI100	Invitrogen (Waltham, MA, USA)
HIV-p24	FITC	KC57	Beckman Coulter (Brea, CA, USA)
HIV-p24	PE	KC57	
HIV-p24	APC	28B7	

PE, phycoérythrine; FITC, Fluorescéine isothiocyanate; APC, Allophycocyanin; BV, Brillant violet

**Supplementary Table 4.** Oliogonucleotides sequence of primers and probes used for HIV-RNA and HIV-DNA quantification

**Primers/Probes**	**Oligonucleotides Sequences**

***Table 4a: External/internal primers and paqman probe used for CA HIV-RNA and DNA quantification***	
ULF1	5'-ATGCCACGTAAGCGAAACTCTGGGTCTCTCTDGTTAGAC-3'
UR1	5'-CCATCTCTCTCCTTCTAGC-3'.
Lambda (λ) T	5'-ATGCCACGTAAGCGAAACT-3'
UR2 IP	5'-CTGAGGGATCTCTAGTTACC-3'.
UHIV FamZen	5'-/56-FAM/CACTCAAGG/ZEN/CAAGCTTTATTGAGGC/3IABkFQ/-3'

***Table 4b: External/internal primers and taqman probe used for CD3 quantification (together with Gag HIV-DNA)***	
HCD3OUT 5' E	5'-ACTGACATGGAACAGGGGAAG-3'
HCD3OUT 3'	5'-CCAGCTCTGAAGTAGGGAACATAT-3'
HCD3IN 5'	GGCTATCATTCTTCTTCAAGGT
HCD3IN3'	CCTCTCTTCAGCCATTTAAGTA
CD3 FamZen	5'-/56FAM/AGCAGAGAA/ZEN/CAGTTAAGAGCCTCCAT/3IABkFQ/-3'

***Table 4c: External/internal primers and FRET probes used for integrated HIV-DNA***	
Alu1	5'-TCCCAGCTACTGGGGAGGCTGAGG-3'
Alu2	5'-GCCTCCCAAAGTGCTGGGATTACAG-3'
Lambda (λ)T	5'-ATGCCACGTAAGCGAAACT-3'
AA55M	5'-GCTAGAGATTTTCCACACTGACTAA-3'
LTRFL	5'-CACAACAGACGGGCACACACTACTTGA-3'-Flurorescein
LTRLC	5'-CACTCAAGGCAAGCTTTATTGAGGC-3'-Phosphate

***Table 4b: External/internal primers and FRET probes used for CD3 quantification (together with integrated HIV-DNA)***	
HCD3OUT5'	5'-ACTGACATGGAACAGGGGAAG-3'
HCD3OUT3'	5'-CCAGCTCTGAAGTAGGGAACATAT-3'
HCD3IN5'	5'-GGCTATCATTCTTCTTCAAGGT-3'
HCD3IN3'	5'-CCTCTCTTCAGCCATTTAAGTA-3'
P1	5'-GGCTGAAGGTTAGGGATACCAATATTCCTGTCTC-3'-Fluorescein
P2	5'-CTAGTGATGGGCTCTTCCCTTGAGCCCTTC-3'-Phosphate
